# Positioning of pivot points in quadrupedal locomotion: limbs global dynamics in four different dog breeds

**DOI:** 10.3389/fbioe.2023.1193177

**Published:** 2023-07-07

**Authors:** Emanuel Andrada, Gregor Hildebrandt, Hartmut Witte, Martin S. Fischer

**Affiliations:** ^1^ Institute of Zoology and Evolutionary Research, Friedrich-Schiller-University Jena, Jena, Germany; ^2^ Group of Biomechatronics, Institute of Mechatronic System Integration, Technische Universität Ilmenau, Ilmenau, Germany

**Keywords:** dog locomotion, leg dynamics, effective legs, VPP-control, dog breed

## Abstract

Dogs (*Canis familiaris*) prefer the walk at lower speeds and the more economical trot at speeds ranging from 0.5 Fr up to 3 Fr. Important works have helped to understand these gaits at the levels of the center of mass, joint mechanics, and muscular control. However, less is known about the global dynamics for limbs and if these are gait or breed-specific. For walk and trot, we analyzed dogs’ global dynamics, based on motion capture and single leg kinetic data, recorded from treadmill locomotion of French Bulldog (*N* = 4), Whippet (*N* = 5), Malinois (*N* = 4), and Beagle (*N* = 5). Dogs’ pelvic and thoracic axial leg functions combined compliance with leg lengthening. Thoracic limbs were stiffer than the pelvic limbs and absorbed energy in the scapulothoracic joint. Dogs’ ground reaction forces (GRF) formed two virtual pivot points (VPP) during walk and trot each. One emerged for the thoracic (fore) limbs (VPP_TL_) and is roughly located above and caudally to the scapulothoracic joint. The second is located roughly above and cranially to the hip joint (VPP_PL_). The positions of VPPs and the patterns of the limbs’ axial and tangential projections of the GRF were gaits but not always breeds-related. When they existed, breed-related changes were mainly exposed by the French Bulldog. During trot, positions of the VPPs tended to be closer to the hip joint or the scapulothoracic joint, and variability between and within breeds lessened compared to walk. In some dogs, VPP_PL_ was located below the pelvis during trot. Further analyses revealed that leg length and not breed may better explain differences in the vertical position of VPP_TL_ or the horizontal position of VPP_PL_. The vertical position of VPP_PL_ was only influenced by gait, while the horizontal position of VPP_TL_ was not breed or gait-related. Accordingly, torque profiles in the scapulothoracic joint were likely between breeds while hip torque profiles were size-related. In dogs, gait and leg length are likely the main VPPs positions’ predictors. Thus, variations of VPP positions may follow a reduction of limb work. Stability issues need to be addressed in further studies.

## 1 Introduction

**FIGURE 1:**
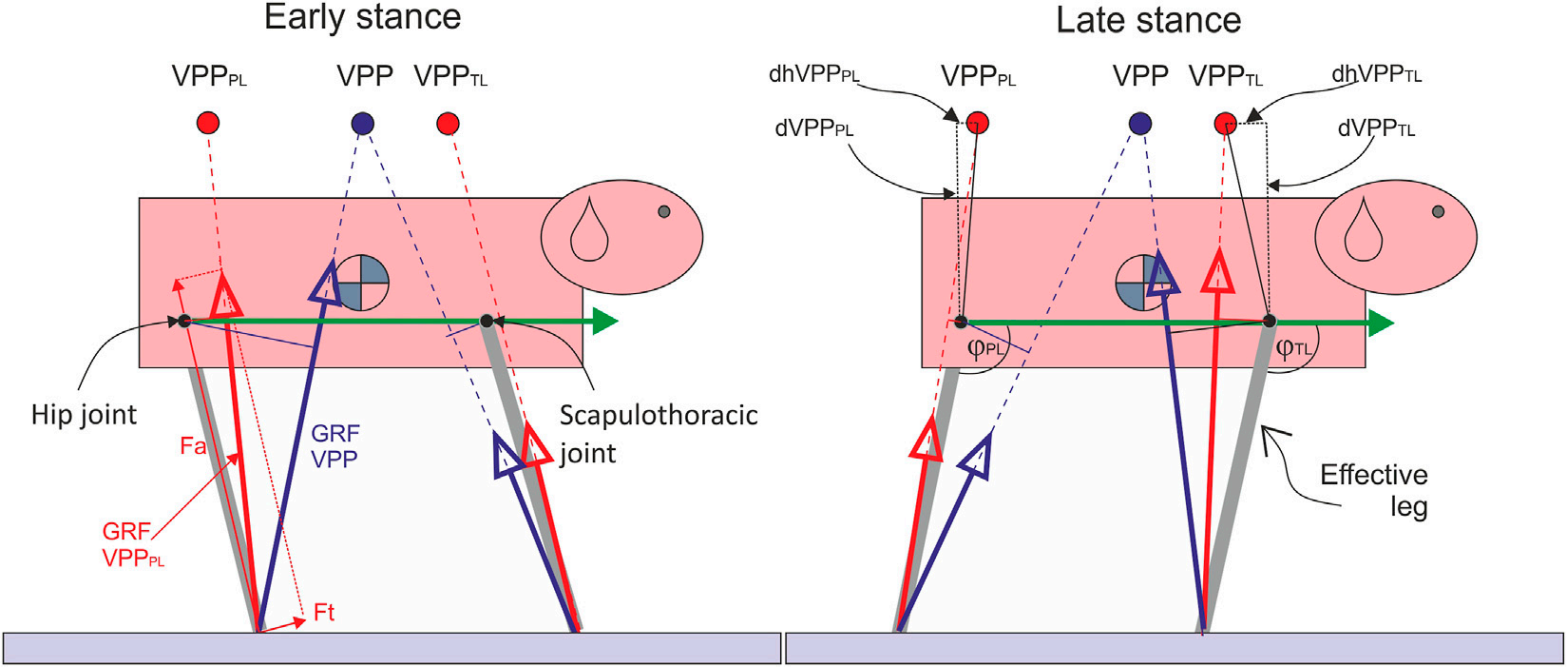
The influence of the number of VPPs on pelvic and thoracic effective leg mechanics. Quadrupeds directing forces to a single VPP above the CoM as observed in bipeds would induce large joint torque and work in the hip and scapulothoracic joints due to the larger lever arms (thin blue solid lines). Moreover, pelvic and thoracic limbs would not act independently. Two proximal joint-related VPPs (VPP_PL_ and VPP_TL_) may solve these problems. The green arrow represents the trunk vector, ϕ_TL_ and ϕ_PL_: angles between the trunk vector and effective legs. Fa: axial force, Ft: tangential force, PL: pelvic limb, TL: thoracic limb, dVPP_PL_: vertical distance VPP_PL_-hip joint, dVPP_PL_: horizontal distance VPP_PL_-hip joint, dVPP_TL_: vertical distance VPP_TL_-scapulothoracic joint (center of scapular rotation in the sagittal plane), and dhVPP_TL_: horizontal distance VPP_PL_-scapulothoracic joint.

Dogs prefer the walk at lower speeds and the (more economical) trot at speeds ranging from 0.5 Fr up to 3 Fr (Jayes and Alexander, 1978; Bryce and Williams, 2017). Fr is a dimensionless measure of speed known as the Froude number (*Fr* = ${v_{t}}^{2}/gl$ ), where *v*_*t*_ is the locomotion speed, *g* is gravitational acceleration, and *l* is the effective leg length (length between most proximal, anatomical limb pivot like the hip joint and the ground contact point, see Figure 1). Walking dogs alternate between a short 2-legged and long 3-legged support of the body; trotting dogs use diagonal pairs of limbs. The different coordination is mirrored in the mechanics of the center of mass, i.e., vaulting mechanics (Cavagna et al., 1977) at walk vs. bouncing mechanics (Blickhan, 1989) at trot. Yet, gait-related differences are more diffuse when looking at the levels of joint dynamics or muscle activations (Tokuriki, 1973a; Tokuriki, 1973b; Andrada et al., 2017; Stark et al., 2021).

Important work has been done to understand dog locomotion at muscular (Tokuriki, 1973a; Tokuriki, 1973b; Tokuriki, 1974; Goslow et al., 1981; Carrier et al., 2006; Carrier et al., 2008; Deban et al., 2012) and at joint levels in healthy and sick dogs (Dogan et al., 1991; Carrier et al., 1998; Gregersen et al., 1998; Nielsen et al., 2003; Colborne et al., 2005; Colborne et al., 2006; Burton et al., 2008; Ragetly et al., 2010; Burton et al., 2011; Colborne et al., 2011; Headrick, 2012; Headrick et al., 2014; Andrada et al., 2017). Still, we know little about the global control of limbs for periodic/stable locomotion.

At the global limb level, the thoracic limbs (fore limbs) have a primordial weight-bearing function and contribute less to propulsion than the pelvic limbs (hindlimbs) (Budsberg et al., 1987; Lee et al., 1999; Bertram et al., 2000; Fischer and Lilje, 2011). Based on the absence of (or extremely low) activity of the main protractor and retractor muscles of the humerus during the stance phase, Carrier and colleagues hypothesized that the thoracic limbs mainly work axially (i.e., as struts at the shoulder joints) (Carrier et al., 2008). This description agrees with the spring-loaded inverted pendulum (SLIP) model. Despite its simplicity, template models like the SLIP model allow to extract key features of quadrupedal locomotion. With such a simple representation, McMahon could explain why galloping is a faster quadrupedal gait than trotting (McMahon, 1985). With a model composed of a rigid torso and prismatic, massless, spring-like effective legs, other authors analyzed the energetics of trotting, bounding, and galloping (Nanua, 1992; Nanua and Waldron, 1995). Later, Poulakakis and colleagues used a similar model to analyze locomotion stability in the sagittal plane (Poulakakis et al., 2003; Poulakakis et al., 2006). Other extensions of those simple models included an articulated torso (Deng et al., 2012; Cao and Poulakakis, 2013). Recently, Söhnel et al. (2020) presented the global leg functions of jumping dogs. They could separate beginner from skilled agility dogs based on SLIP-related parameters.

Because of its explaining power, the behavior of the effective leg is often separated into two main time-related functions: an axial and a tangential or rotational leg function (Maus et al., 2010; Shen and Seipel, 2012; Andrada et al., 2014). The axial leg function combines the axial force (*F*_*a*_) with the length change of the instantaneous effective leg (*F*_*a*_ vs. Δ*l*) relative to the leg length at touch down (TD) (*l*_0_). Fa is the component of the ground reaction forces (GRF) along the effective leg (axial). The axial leg function provides mainly a weight-bearing function, generates vertical body oscillations, and is usually represented by leg stiffness or, more exactly, leg impedance.

The tangential leg function can be displayed by combining the proximal torque with the joint angle **(** *M* vs. ϕ**)**. The proximal joint torque *M* is obtained by multiplying the force perpendicular to the effective leg (*F*_*t*_, therefore tangential function) by the instantaneous effective leg length *l*. The tangential leg function represents the strategy used to retract the effective leg and balance the trunk. The vectorial sum of *F*_*a*_ and *F*_*t*_ yields the vector of GRF, which we measure via force plates (see Figure 1).

To generate periodic locomotion, both axial and tangential leg functions must be combined in a way that leg retraction matches the oscillation time along the leg. In bipeds, the trunk must be additionally balanced. The combination of experimental and simulation studies has shown that a simple strategy of directing GRF to a body-fixed point above the CoM can be used to balance the trunk and generate coordination between both leg functions, leading to stable gaits (Maus et al., 2010; Andrada et al., 2014; Drama and Badri-Spröwitz, 2020). This body-fixed point was termed Virtual Pivot Point (VPP).

In difference to bipeds, dogs display two body-fixed VPPs during steady-state locomotion: one above the hip and another above the shoulder (Figure 1). Jayes and Alexander (1978, p. 304 and Figure 12) first described VPPs as “the points through which the forces on the fore and hind feet tend to act” in dogs and a sheep without naming it explicitly.

Surprisingly, the dynamic implications of having two VPPs and their relation to the effective leg’s axial and tangential functions in quadruped locomotion remain, until these days, largely unexplored. Furthermore, it is unknown whether the VPPs’ positions and effective leg functions are specific for gait or, in the case of dogs, even breeds.

In the present paper, we analyze the global dynamics of four dog breeds that differ in body mass, posture, and breed purpose during walk and trot. We expected that, after accounting for mass and length measures, the global dynamics represented by VPP position and the axial and tangential leg functions will be similar among different dog breeds for the same gait. If so, then differences in limb segmental kinematics, e.g., (Fischer and Lilje, 2011; Fischer et al., 2018), might reflect adaptations to body, limb proportions, and posture. On the other hand, breed-related differences in the position of the VPPs and/or in the leg axial and tangential leg functions at the same gait may inform limb global dynamic adaptations related to body proportions, posture, and behavior.

Finally, we awaited gait-related changes in global dynamics. At the level of the axial leg function, we expected changes due to the differences between vaulting and bouncing mechanics. For the VPP, we expected that it should be located closer to the most proximal joint for trot to reduce joint torque/work in the most proximal joint.

## 2 Materials and methods

### 2.1 Animals

In the present work, we recomputed part of the data used by Andrada et al. (2017) for Fischer et al. (2018) and included unpublished kinematic and kinetic data for the thoracic limbs of Malinois, French Bulldog, Whippet, and Beagle. Animal details were published in previous works and will be only briefly summarized here: we collected data from five adult male Beagles belonging to a research colony based at the Small Animal Hospital of the University of Veterinary Medicine, Hannover, Germany; four adult Malinois (three males/one female) kept as police dogs by the Saxonian police force; four adult female French Bulldogs from private dog owners; and five adult Whippets (two males/three females) from private dog owners. Table 1 describes the available individuals.

**TABLE 1:** Dogs and the number of steps analyzed for this study.

Breed	Individual	W [kg]	HW [m]	*l*_0_		Steps walk		Steps trot	
				Walk [PL, TL] [m]	Trot [PL, TL] [m]	PL	TL	PL	TL
Beagle	Erwin	14.9	0.35	[0.34, 0.36]	[0.35, 0.35]	10	10	13	12
	Simon	13.8	0.33	[0.32, 0.35]	[0.32, 0.34]	15	16	9	8
	Malte	14.8	0.34	[0.32, 0.36]	[0.32, 0.36]	26	26	32	31
	Louis	16.2	0.38	[0.35, 0.34]	[0.36, 0.35]	21	14	21	21
	Spencer	19.8	0.42	[0.43, 0.45]	[0.43, 0.43]	12	11	29	13
Malinois	Zora	28.5	0.64	[0.53, 0.54]	[0.51, 0.57]	9	12	9	5
	Pike	28.5	0.62	[0.53, 0.54]	[0.53, 0.54]	9	10	5	8
	Hunter	18.6	0.59	[0.48, 0.52]	[0.47, 0.51]	10	10	10	7
	Rocky	22.4	0.58	[0.50, 0.56]	[0.52, 0.53]	7	10	10	8
French Bulldog	Queny	11	0.31	[0.27, 0.26]	[0.28, 0.26]	15	17	20	15
	MJ	9.5	0.30	[0.23, 0.26]	[0.24, 0.25]	17	16	18	7
	Chacha	10	0.31	[0.26, 0.26]	[0.27, 0.26]	19	19	18	8
	Juno	13	0.32	[0.27, 0.28]	[0.28, 0.28]	20	16	19	19
Whippet	Lilly	12	0.49	[0.40, 0.43]	[0.40, 0.43]	18	19	19	5
	Kenja	10	0.46	[0.39, 0.44]	[0.40, 0.43]	4	3	21	20
	Africa	9	0.47	[0.41, 0.43]	[0.42, 0.42]	16	15	13	41
	Merlin	16.5	0.51	[0.44, 0.45]	[0.45, 0.47]	18	17	10	6
	Moody	13.3	0.50	[0.42, 0.46]	[0.42, 0.44]	17	20	12	6

W, weight; HW, height at the withers; *l*_0_, leg length at TD (mean value); PL, pelvic limb; TL, thoracic limb.

### 2.2 Marker setup, motion analysis, and kinetics

The marker setup encompassed 19 markers on the left thoracic limb and 21 markers on the left pelvic limb. For the purpose of this work, we used only the most proximal and distal leg markers to describe both the pelvic and the thoracic effective legs and trunk. For the pelvic limb, the effective leg was computed as the direct connection between the marker placed on the dorsal aspect of the third metatarsal bones and the marker located at the greater trochanter of the femur. The thoracic limb effective leg was computed as the distance between the dorsal aspect of the third phalanx and the scapulothoracic joint. Based on our previous works, we assumed that the scapulothoracic joint is located at 2/3 of the distance between the markers representing the most dorsal and ventral points of the scapula (Fischer, 1999; Andrada et al., 2017).

3D kinematic data were collected using 6 infrared Vicon^®^ cameras (Oxford Metrics, Oxford, United Kingdom) and an instrumented quad-band treadmill (model 4060-08, Bertec Corporation) available at the locomotion lab of the Small Animal Hospital of the University of Veterinary Medicine, Hannover, Germany. Kinematic data was collected at 100 Hz and force data at 1,000 Hz. Belt speed was controlled by using the Bertec treadmill control panel, v. 1.7.12. Data collection started as soon as the dogs were walking or trotting smoothly and comfortably. Data was recorded for a maximum of 45 s. For computation, a series of at least five cycles (strides) in which the dog moved steadily and without overstepping onto the other bands (force plates) were used. When trotting, dogs were kept on one side of the treadmill (usually the left side) to facilitate handling. The computed number of steps can be found in Table 1. The lab coordinate system was set as follows: +x pointed left, +y pointed opposite to the direction of motion, and +z pointed upwards. The 3D coordinates of marker trajectories were smoothed by a Butterworth four-order low-pass filter with a cut-off frequency of 6 Hz applied in a zero-phase digital filter. Force data were down-sampled to 100 Hz to cope with kinematical data and posteriorly filtered using a 7th-order Butterworth low-pass filter with a cut-off frequency of 20 Hz.

In this work, we used the sagittal projection of both kinematics and GRF. To describe the axial leg function, we combined the instant changes of effective leg length ∆l and axial force $\vec{F_{a}}$ during stance. $\vec{F_{a}}$ was computed by projecting the vector of the GRF into the vector defining the effective leg $\vec{l}$ (see Figure 1). The tangential function combines joint torque M and effective leg angle ϕ relative to the trunk vector. The trunk vector was defined as a vector between the hip joint and the scapulothoracic joint. The angle ϕ between the trunk vector and effective leg was computed using the dot product between two vectors (see Figure 1). Proximal joint torque was computed as $M=\left|\vec{F_{t}}\right|.\left|\vec{l}\right|,$ where the tangential force $\vec{F_{t}}$ was previously obtained by computing the component of the vector of the GRF perpendicular to $\vec{l}$ (see Figure 1). VPP and paw positions were computed relative to the proximal joints (hip and scapulothoracic joints), adapting the methods proposed for the CoM in Andrada et al. (2014). For more details, see the Supplementary Material. Note that for each limb pair, we described the relative position of the distal point of the effective legs and the direction of the GRF during the stance from a moving frame. Therefore, although the scapulothoracic joint translates relatively to the trunk during stance in the global coordinates, in the relative coordinates we used, it is a fixed point. In the plots, the positions of the proximal joints were frozen at their values at TD. For the sake of comparison, we transformed force, length, and torque into a nondimensional form*.* For the force, we used $\hat{F}=F/mg$ *;* for the length $\hat{l}=\frac{l}{l_{0}}$ , $l_{0}$ being the effective leg length at TD; for the change of effective leg length $∆\hat{l}=\frac{l}{l_{0}}-1$ ; and for the torque $\hat{T}=T/mgl_{0}$ . Axial work was computed as the area inside the loop in the graph ${\hat{F}}_{a}$ vs. $∆\hat{l}$ , while tangential work as the area below the curve $\hat{T}$ vs. ϕ. We modeled the axial effective leg function as a parallel spring-damper model, ${\hat{F}}_{am}=\hat{k}\left(∆\hat{l}\right)-\hat{c}∆\dot{\hat{l}}$ (Andrada et al., 2014) to obtain dimensionless stiffness and damping coefficients. ${\hat{F}}_{am}$ is the axial force computed by the model, $\hat{k}$ is the dimensionless effective leg stiffness, $\hat{c}$ is the dimensionless effective leg damping, and $∆\dot{\hat{l}}$ the rate of change of effective leg length. We obtained $\hat{k}$ and $\hat{c}$ by using a nonlinear fit that minimized for each trial the sum of squared distances between measured forces ${\hat{F}}_{a}$ and the forces ${\hat{F}}_{am}$ calculated with the spring-damper model (using $∆\hat{l},$ $∆\dot{\hat{l}}$ from experiments). Note that our results showed that the effective leg in the dog can dissipate or generate energy axially during locomotion. Therefore, we permitted the damping coefficient $\hat{c}$ to take positive (dissipate energy) or negative (generate energy) values when fitting the experimental axial leg function. The relationships between dimensionless and dimensional stiffness and damping are: $\hat{k}=\frac{kl_{0}}{mg}$ , $\hat{c}=\frac{c}{m}\sqrt{\frac{l_{0}}{g}}$ . Global dynamics were computed using custom-written scripts in Matlab^®^ 2017 (The MathWorks^®^ Inc., Natick, MA, United States).

### 2.3 Statistical analysis

To infer the influence of gait and breed on the vertical (dVPP) and horizontal (dhVPP) positions of the VPP measured from the hip and scapulothoracic joint, maximal axial force ${\hat{F}}_{a-\max}$ , maximal joint Torque *M*_max_, leg angle relative to the trunk at TD (ϕ_0_), leg length at toe-off (TO), and $\hat{k}$ and $\hat{c}$ , repeated measures ANOVA with Gait (walk vs. trot) as within-subjects and Breed as between subjects were performed. Afterward, Post hoc tests with Bonferroni correction were performed for significant breed dependencies (*p* < 0.05). Statistical analysis was performed in IBM^®^ SPSS^®^ Statistics 26.

## 3 Results

During data collection, the dogs’ mean speed ±SD at walk (w) and trot (t) were: Malinois: (w: 1.2 ± 0 m/s, t: 2.5 ± 0.3 m/s); Whippet: (w: 1.0 ± 0.05 m/s, t: 1.8 ± 0.21 m/s); French Bulldog: (w. 0.8 ± 0.05 m/s, t: 1.5 ± 0.11 m/s); Beagle: (w. 1.0 ± 0.04 m/s, t: 2.2 ± 0.22 m/s).

**FIGURE 2:**
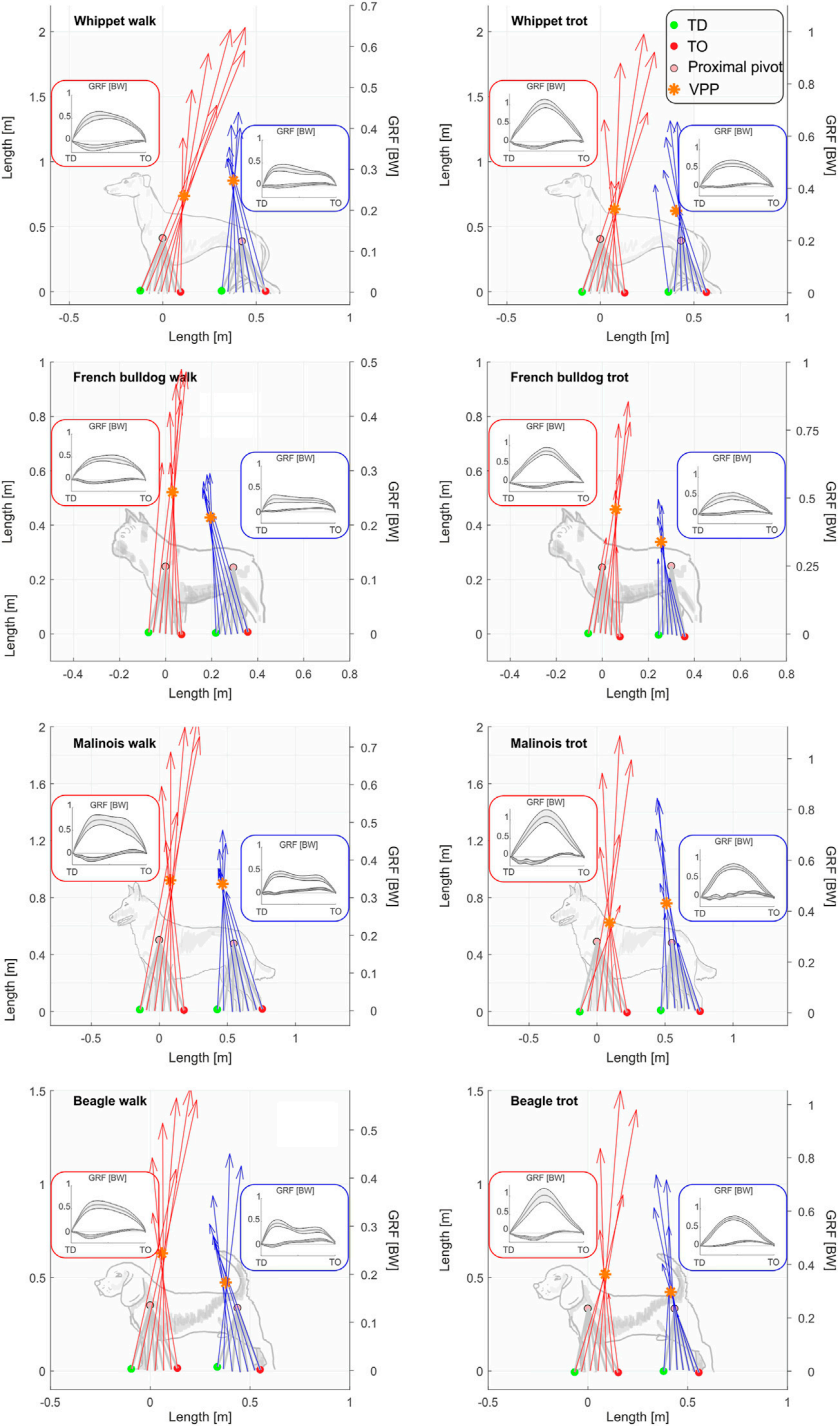
The two VPPs are still evident even when averaging data from all individuals of a breed. Subplots show mean VPP_TL_ and VPP_PL_ for all analyzed breeds at walk and trot. Ground reaction forces and leg orientation are mean values for all individuals and strides of each bread. The red arrows correspond to the mean GRF of the thoracic limbs, while the blue arrows correspond to the mean GRF of the pelvic limbs. Distal points of the legs and GRF were computed relative to the proximal joints. Therefore, the proximal joints can be displayed as fixed points (see methods). Superimposed squares display means and SD of the vertical and fore-aft components of the GRF in BW. Superimposed dog sketches were included for an easier interpretation of the figures. They were not isometrically scaled. Beagle (*N* = 5), strides walk = 84 for pelvic limbs and 77 for the thoracic limbs; strides trot = 104 for pelvic limbs and 85 for the thoracic limbs. French Bulldog (*N* = 4), strides walk = 71 for pelvic limbs and 68 for the thoracic limbs; strides trot = 75 for pelvic limbs and 49 for the thoracic limbs. Malinois (*N* = 4), strides walk = 35 for pelvic limbs and 42 for the thoracic limbs; strides trot = 34 for pelvic limbs and 28 for the thoracic limbs. Whippet (*N* = 5), strides walk = 73 for pelvic limbs and 74 for the thoracic limbs; strides trot = 75 for pelvic limbs and 78 for the thoracic limbs. GRF profiles for each breed can be found in the Supplementary Figures S3–S6.

All breeds displayed a proximal joint-related VPP point above both the hip and the scapulothoracic joints (center of scapular rotation in the sagittal plane) during walk (Figure 2). As hypothesized, at trot, the distance from the VPP to the proximal pivot was significantly decreased. For three Whippets and one Malinois, the VPP for the pelvic limb was found to be even closely below the hip. In addition, our results show that leg function is rather similar among different dog breeds, but for French Bulldogs and Whippets, some deviations were found.

### 3.1 Pelvic limb and axial leg function

**FIGURE 3:**
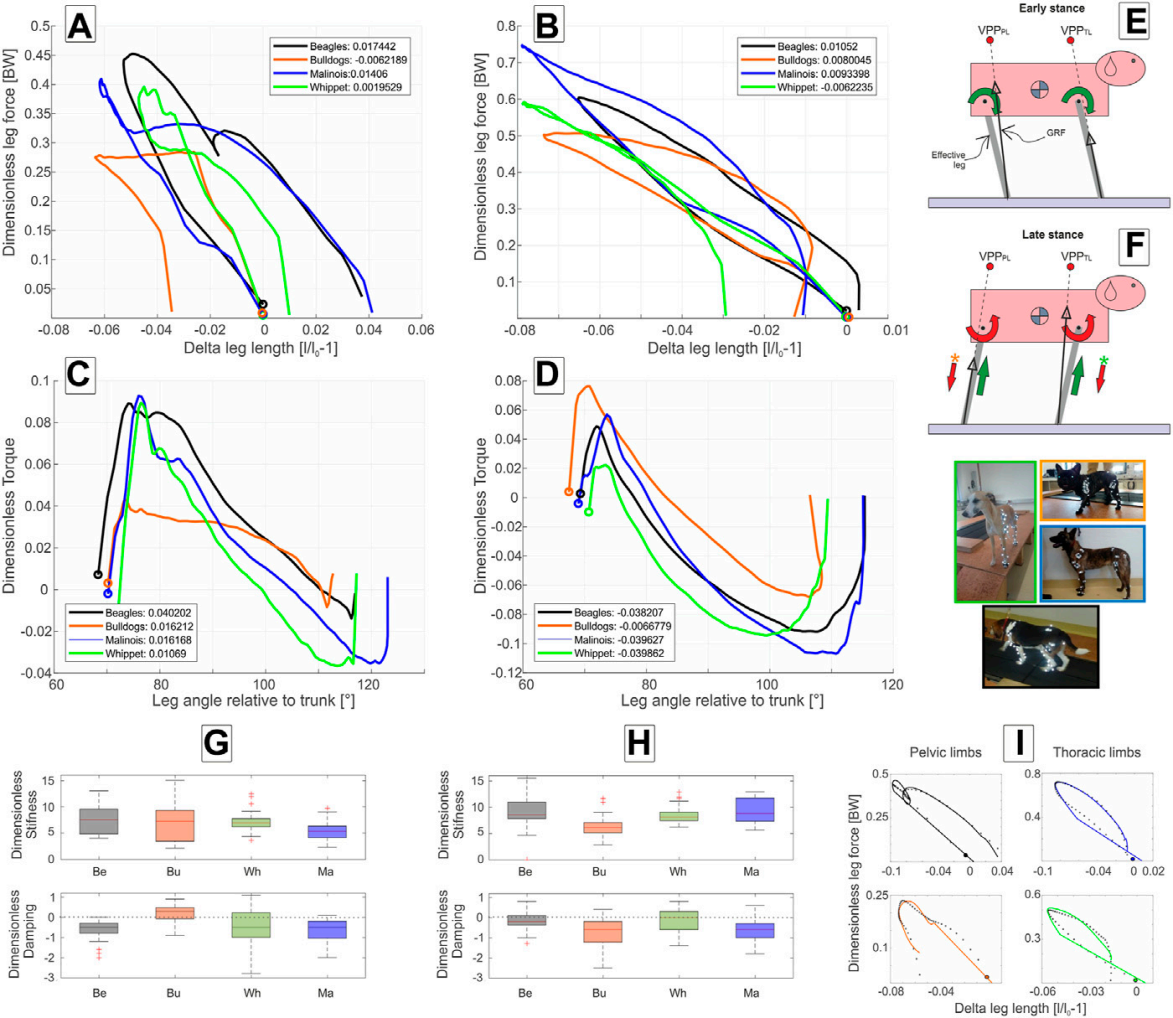
Axial and tangential leg functions at walk-in Beagle (black), Whippet (green), French Bulldog (orange), and Malinois (blue). **(A,C)** pelvic limb; **(B,D)** thoracic limb. **(A,B)** axial function; **(C,D)** tangential function and **(G,H)** stiffness and damping of the effective leg. Values are dimensionless. The curves in **(A–D)** represent mean values. In **(A–D,I)**, circles indicate TD. Positive values in **(C,D)** represent retractors while negative represent protractor torques. Legends show net axial/tangential work. **(E,F)** template representation at early and late stance based on the curves in **(A–D)**. The position of the VPPs and leg orientations are rough approximations. For more accurate positions, see Figure 2; Table 5. In **(E,F)**, the curved arrows represent torque direction. Linear arrows indicate leg extension/shortening. The green arrows indicate energy generation (motions and force/torque directions coincide) and the red indicates energy absorption (motions and force/torque directions are opposite). Note that F arrows with (*) display generalized leg functions in French Bulldog and Whippet that differed from the two other breeds. Negative damping in G or H indicates axial energy generation, while positive damping indicates axial energy dissipation in the effective leg. **(I)** examples of axial leg function from experiments (black asterisks) and results of the nonlinear fit using a parallel spring-damper model (colored line). Larger figure versions of **(G–I)** can be found in the Supplementary Figures S7, S9.

At walk, the axial leg function diverges from the pure spring-like leg behavior during stance. In mean, $\hat{k}$ was around 7 for Beagles, Bulldogs, and Whippets, and $\hat{k}$ ≈ 5.5 for Malinois. Beagles and Malinois displayed similar leg functions. Both exhibited effective leg lengthening of approximately 4% with respect to the length measured at TD, and, correspondingly, negative $\hat{c}$ values (−0.6 and −0.7, for Beagles and Malinois, respectively). Contrarily, in French Bulldogs, leg length was shortened during stance to approximately the same amount (leading to positive $\hat{c}$ values around 0.2). A picture closer to a spring-like leg behavior was observed for Whippets although they still showed small leg lengthening and negative $\hat{c}$ mean values around −0.5 (Figure 3G; Table 4).

**FIGURE 4:**
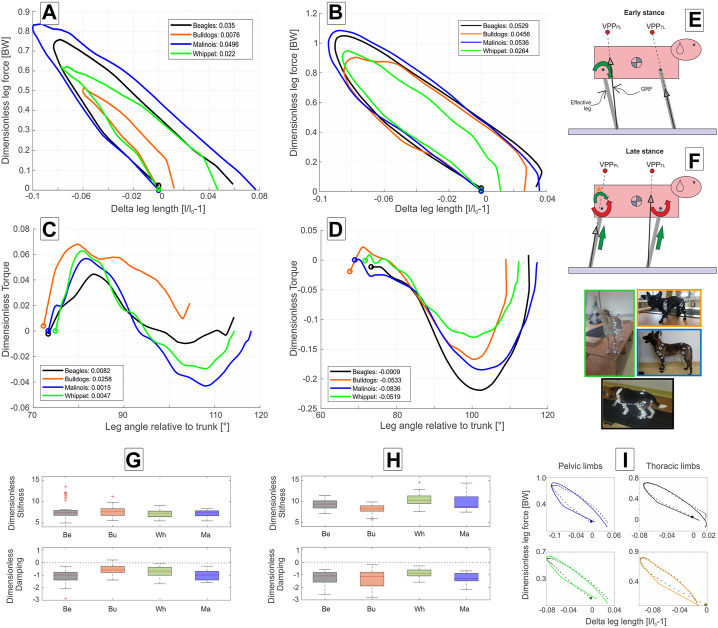
Axial and tangential leg functions at trot in Beagle (black), Whippet (green), French Bulldog (orange), and Malinois (blue). **(A,C,G)** pelvic limb; **(B,D,H)** thoracic limb. **(A,B)** axial function; **(C,D)** tangential function, and **(G,H)** stiffness and damping of the effective leg. Values are dimensionless. The curves in **(A–D)** represent mean values. The circles indicate TD. Positive values in **(C,D)** represent retractors while negative represent protractor torques. Legends show net axial/tangential work. **(E,F)** template representation at early and late stance based on the curves **(A–D)**. Position of the VPPs and leg orientations are rough approximations and may vary between breeds. For accurate data, see Figure 2; Table 5. In **(E,F)**, curved arrows represent torque direction. Linear arrows indicate leg extension/shortening. Green arrows indicate energy generation (motions and force/torque directions coincide), and red energy absorption (motions and force/torque directions are opposite). Note that in **(F)**, the curved arrow with (*) displays tangential leg function in the French Bulldog that differed from the mainstream. Negative damping in **(G)** or **(H)** indicates axial energy generation, while positive damping indicates axial energy dissipation in the effective leg. **(I)** examples of axial leg function from experiments (black asterisks) and results of the nonlinear fit using a parallel spring-damper model (colored lines). Larger figure versions of Figures 4G–I can be found in the Supplementary Figures S8, S10.

At trot, all four breeds exhibited larger ${\hat{F}}_{a-\max}$ (*p* < 0.001) and leg lengthening during the late stance phase (*p* < 0.001) than during walk. Leg lengthening induced larger negative $\hat{c}$ values than were observed at walk (Figures 4A, G; Tables 2, 4). Again, Beagles and Malinois displayed similar leg functions. They showed the highest projected leg force ( ${\hat{F}}_{a-\max}$ > 0.7) and leg lengthening between 6% and 7%, and thus the highest positive axial work (see Figure 4A). Whippets displayed lower peak axial forces and less leg lengthening than Malinois and Beagles. French Bulldogs displayed the lowest peak axial force (approximately 0.5 BW), and a more spring-like leg behavior (damping coefficient close to zero, see Figure 4G). Therefore, they exerted the lowest positive net axial work.

**TABLE 2:** Pelvic limb: components of the axial (Fa_PL_ vs. Δl_PL_) and the tangential leg function (*M*_PL_ vs. ϕ_PL_)

% Of stance time	1	25	50	75	99
Beagle					
Δ*l*_PL_ (w)	−0.01 ± 0	−0.05 ± 0.04	−0.02 ± 0.04	−0.02 ± 0.03	0.03 ± 0.03
Δ*l*_PL_ (t)	−0.003 ± 0	−0.071 ± 0.01	−0.074 ± 0.02	−0.010 ± 0.013	0.057 ± 0.01
Fa_PL_ (w)	0.06 ± 0	0.45 ± 0.03	0.29 ± 0.03	0.32 ± 0.03	0.06 ± 0.02
Fa_PL_ (t)	0.042 ± 0.001	0.552 ± 0.009	0.747 ± 0.016	0.426 ± 0.013	0.044 ± 0.009
ϕ_PL_ (w) [°]	68.9 ± 1.4	80.1 ± 2.7	92.1 ± 3.1	106.6 ± 3.5	116.7 ± 3.5
ϕ_PL_ (t) [°]	73.7 ± 1.5	85.3 ± 1.7	97.6 ± 2.5	106.8 ± 4.1	114.0 ± 4.2
*M*_PL_ (w)	0.020 ± 0.005	0.085 ± 0.037	0.043 ± 0.021	0.009 ± 0.015	−0.007 ± 0.009
*M*_PL_ (t)	0.001 ± 0.006	0.041 ± 0.014	0.001 ± 0.022	−0.005 ± 0.013	0.009 ± 0.008
French Bulldog					
Δ*l*_PL_ (w)	−0.003 ± 0	−0.028 ± 0.02	−0.053 ± 0.026	−0.062 ± 0.033	−0.036 ± 0.04
Δ*l*_PL_ (t)	−0.002 ± 0	−0.047 ± 0.01	−0.058 ± 0.014	−0.024 ± 0.01	0.011 ± 0.012
Fa_PL_ (w)	0.04 ± 0	0.28 ± 0.02	0.28 ± 0.03	0.27 ± 0.03	0.05 ± 0.033
Fa_PL_ (t)	0.03 ± 0	0.40 ± 0.01	0.51 ± 0.01	0.36 ± 0.01	0.03 ± 0.014
ϕ_PL_ (w) [°]	70.5 ± 3.8	76.5 ± 3.6	87.3 ± 2.6	100.8 ± 1.74	112.5 ± 3.6
ϕ_PL_ (t) [°]	72.6 ± 3.2	81.0 ± 3	89.9 ± 2.9	97.9 ± 2.6	104.3 ± 2.9
*M*_PL_ (w)	0.010 ± 0.027	0.038 ± 0.106	0.033 ± 0.074	0.022 ± 0.034	0.005 ± 0.008
*M*_PL_ (t)	−0.012 ± 0.012	−0.075 ± 0.014	−0.218 ± 0.01	−0.144 ± 0.01	−0.002 ± 0.006
Malinois					
Δ*l*_PL_ (w)	−0.006 ± 0	−0.061 ± 0.025	−0.052 ± 0.02	−0.028 ± 0.031	0.040 ± 0.028
Δ*l*_PL_ (t)	−0.004 ± 0	−0.085 ± 0.013	−0.09 ± 0.02	−0.011 ± 0.02	0.075 ± 0.019
Fa_PL_ (w)	0.05 ± 0	0.41 ± 0.021	0.33 ± 0.022	0.33 ± 0.06	0.04 ± 0.02
Fa_PL_ (t)	0.03 ± 0	0.65 ± 0.01	0.83 ± 0.02	0.51 ± 0.022	0.02 ± 0.01
ϕ_PL_ (w) [°]	70.9 ± 3.8	83.3 ± 4.7	97.0 ± 5.2	113.0 ± 5.1	123.0 ± 7.5
ϕ_PL_ (t) [°]	73.8 ± 3.2	86.4 ± 3.5	100.1 ± 4.1	111.1 ± 5.2	117.9 ± 5.7
*M*_PL_ (w)	0.012 ± 0.015	0.063 ± 0.028	0.016 ± 0.023	−0.021 ± 0.024	−0.008 ± 0.013
*M*_PL_ (t)	0.007 ± 0.012	0.046 ± 0.014	−0.025 ± 0.01	−0.036 ± 0.01	−0.002 ± 0.006
Whippet					
Δ*l*_PL_ (w)	−0.004 ± 0	−0.045 ± 0.02	−0.044 ± 0.026	−0.030 ± 0.022	0.009 ± 0.033
Δ*l*_PL_ (t)	−0.003 ± 0	−0.063 ± 0.011	−0.073 ± 0.021	−0.021 ± 0.026	0.045 ± 0.025
Fa_PL_ (w)	0.06 ± 0	0.39 ± 0.02	0.32 ± 0.021	0.29 ± 0.01	0.05 ± 0.03
Fa_PL_ (t)	0.03 ± 0	0.55 ± 0.01	0.62 ± 0.05	0.45 ± 0.02	0.03 ± 0.02
ϕ_PL_ (w) [°]	72.6 ± 3.1	82.8 ± 3.2	93.5 ± 3.3	106.1 ± 5	117.0 ± 5.6
ϕ_PL_ (t) [°]	75.3 ± 2	85.6 ± 3	97.6 ± 4.2	108.0 ± 5	114.2 ± 4.4
*M*_PL_ (w)	0.005 ± 0.016	0.055 ± 0.012	0.014 ± 0.014	−0.023 ± 0.023	−0.008 ± 0.005
*M*_PL_ (t)	0.007 ± 0.007	0.048 ± 0.022	−0.010 ± 0.035	−0.030 ± 0.030	−0.001 ± 0.009

Mean values ±SD, for the axial force (Fa_PL_, Fa/*mg*), the leg length change (Δl_PL_ = *l/l*_0_-1), the angle between leg and trunk vector (ϕ_PL_), and the hip torque (*M*_PL_) at walk (w) and trot (t). Note that Fa_PL_, Δl_PL,_ and *M*_PL_, are dimensionless. *l* is leg length and *l*_0_ leg length at TD; m, mass and g, gravity.

In mean, $\hat{k}$ values were around 7 for all breeds, ranging from $\hat{k}$ = 7.1 (Whippets) to $\hat{k}$ = 7.9 (Beagles). The dimensionless leg stiffness ( $\hat{k}$ ) was gait (*p* < 0.01), breed (*p* = 0.005), and gait * breed-related (*p* = 0.02). However, this finding is only explained by the more compliant pelvic limb exhibited by Malinois at walk. By excluding Malinois from the ANOVAs, $\hat{k}$ , gait or breed-related changes vanished. The leg-lengthening was gait-related (*p* < 0.001) but not breed-related.

The dimensionless leg damping ( $\hat{c}$ ) was also gait, breed, and gait * breed related (all three *p* < 0.001). In this study, breed-related changes are explained by a different pelvic limb control strategy in the French Bulldog (see also Sections 3.2 and 3.3).

### 3.2 Pelvic limb and tangential leg function

At walk, the angle of attack ϕ_0_ is at approximately 70° in all four breeds, and the lift-off angle ϕ between 112° (French Bulldog) and 123° (Malinois), see Table 2. During most of the stance phases, the hip muscles actively retract the pelvic limb (positive torque and leg retraction; Figure 3C).

Maximal positive peak torque was similar in time and value (T∼ 0.09) for Whippets, Malinois, and Beagles, but approximately half of that value for French Bulldogs. The mean torque profile was basically biphasic. However, only Malinois and Whippets displayed a markedly negative torque (leading to protraction) in the late stance phase.

During trot, effective legs touched the ground a steeper than at walk (about 74°, *p* < 0.001). Effective legs were between 3° and 8° less retracted during stance at trot compared to walk (*p* < 0.05, see Table 2). Mean torque profiles were biphasic for all analyzed breeds except for French Bulldogs, which showed a half sinus profile (Figure 4C). Maximal torques were gait-related (*p* < 0.05). French Bulldogs also displayed the largest peak positive torque. For the other breeds, the maximal positive (retractor) torques were lower than those exhibited during walk.

### 3.3 Pelvic limbs and VPP

**FIGURE 5:**
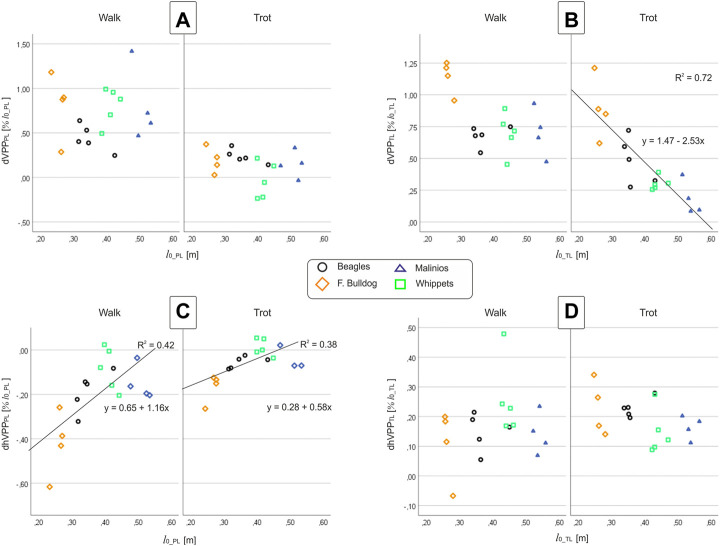
**(A,B)** vertical VPP and **(C,D)** horizontal VPP distances to the proximal joints vs. leg length at TD at walk and trot. Left column **(A,C)**: pelvic limb (PL), right column **(B,D)**: thoracic limb (TL). Points represent the mean value for one dog. While dVPP_PL_ and dhVPP_TL_ do not display any correlation with breed or *l*_0_, dVPP_TL_ and dhVPP_PL_ exhibit dependencies on *l*_0_.

The position of the pelvic limbs’ VPP (VPP_PL_) is gait-related. The VPP_PL_ point was significantly (*p* < 0.001) higher placed at walk (approximately 0.5 and 1.0 of leg length at TD) compared to its position at trot (in mean around 0.2 of leg length at TD above the pelvis for Beagle, French Bulldog, and Malinois and −0.03 below this proximal joint for the Whippets). However, the distance between VPP_PL_ and hip joint did not significantly vary between breeds nor did the linear combination of gait effects * breed effects (see Table 5). Further analyses indicate that the vertical position of the VPP_PL_ was only influenced by gait while their horizontal position was influenced by leg length and gait (see Figure 5).

### 3.4 Thoracic limb and axial leg function

Except for French Bulldogs, the thoracic limbs were stiffer than the pelvic limbs during walk. Beagles and Malinois displayed similar values $\hat{k}$ ≈ 9. Whippets and French Bulldogs exhibited relatively more compliant legs ( $\hat{k}$ ≈ 8 and $\hat{k}$ ≈ 6, respectively). For Whippets and Beagles, $\hat{c}$ oscillated around zero (see Figure 3H; Table 4). In Whippets, only the last 10% of the stance diverged from a worthy spring-like leg and finished with a leg shortening of approximately 3% of *l*_0_ and negative axial work. The other breeds, even when showing very small leg lengthening or shortening, produced positive net axial work (see Figure 3B; Table 3). At trot, all breeds displayed similar axial leg functions and exhibited leg enlargement (between 1.1% for Whippets, 2.7% for French Bulldogs, and approximately 3.5% for Beagles and Malinois). Consequently, all breeds generated positive net axial work and displayed roughly similar viscoelastic parameters $\hat{k}$ and $\hat{c}$ (see Figure 4B and Table 4). Gait influenced leg length at TO and ${\hat{F}}_{a-\max}$ (both *p* < 0.001). Gait and breed significantly influenced $\hat{k}$ and $\hat{c}$ (both *p* < 0.01). The linear combination gait * breed was likewise significant ( $\hat{k}$ : *p* = 0.02, $\hat{c}$ : *p* < 0.01).

**TABLE 3:** Thoracic limb: components of the axial (Fa_TL_ vs. Δl_TL_) and of the tangential leg function (*M*_TL_ vs. ϕ_TL_)

% Stance phase	1	25	50	75	99
Beagle					
Δl_TL_ (w)	−0.004 ± 0	−0.064 ± 0.01	−0.055 ± 0.026	−0.034 ± 0.035	0.003 ± 0.04
Δl_TL_ (t)	−0.004 ± 0	−0.077 ± 0.01	−0.079 ± 0.014	−0.005 ± 0.015	0.035 ± 0.02
Fa_TL_ (w)	0.06 ± 0	0.58 ± 0.01	0.57 ± 0.02	0.43 ± 0.03	0.06 ± 0.05
Fa_TL_ (t)	0.05 ± 0	0.67 ± 0.01	1.04 ± 0.01	0.53 ± 0.01	0.05 ± 0.02
ϕ_TL_ (w) [°]	69.6 ± 3.4	80.3 ± 1.7	96.7 ± 1.8	108.5 ± 2	115.4 ± 2.9
ϕ_TL_ (t) [°]	73.8 ± 5.3	86.6 ± 4.4	100.8 ±	111.5 ± 3.3	115 ± 3
*M*_TL_ (w)	0.01 ± 0.01	−0.02 ± 0.03	−0.08 ± 0.04	−0.09 ± 0.03	−0.01 ± 0.03
*M*_TL_ (t)	−0.012 ± 0.01	−0.075 ± 0.003	−0.218 ± 0.05	−0.144 ± 0.028	−0.002 ± 0.01
French Bulldog					
Δl_TL_ (w)	−0.004 ± 0	−0.063 ± 0.014	−0.066 ± 0.029	−0.032 ± 0.036	−0.012 ± 0.035
Δl_TL_ (t)	−0.004 ± 0	−0.076 ± 0.01	−0.072 ± 0.02	−0.011 ± 0.024	0.027 ± 0.024
Fa_TL_ (w)	0.05 ± 0	0.45 ± 0.01	0.51 ± 0.08	0.42 ± 0.03	0.05 ± 0.03
Fa_TL_ (t)	0.04 ± 0	0.63 ± 0.01	0.89 ± 0.02	0.55 ± 0.02	0.03 ± 0.02
ϕ_TL_ (w) [°]	67.8 ± 5	77.5 ± 4.4	92.4 ± 3.8	104.6 ± 3.5	106.9 ± 5.1
ϕ_TL_ (t) [°]	68.2 ± 5	82.0 ± 5.4	95.5 ± 4.7	105.3 ± 4.2	109.0 ± 2.8
*M*_TL_ (w)	0.024 ± 0.007	0.032 ± 0.032	−0.035 ± 0.036	−0.067 ± 0.032	−0.011 ± 0.005
*M*_TL_ (t)	−0.01 ± 0.01	−0.03 ± 0.04	−0.15 ± 0.09	−0.13 ± 0.06	−0.01 ± 0.01
Malinois					
Δl_TL_ (w)	−0.004 ± 0	−0.074 ± 0.01	−0.073 ± 0.004	−0.043 ± 0.01	−0.010 ± 0.023
Δl_TL_ (t)	−0.004 ± 0	−0.083 ± 0.005	−0.084 ± 0.006	−0.019 ± 0.006	0.036 ± 0.009
Fa_TL_ (w)	0.05 ± 0	0.70 ± 0.01	0.70 ± 0	0.55 ± 0.01	0.05 ± 0.02
Fa_TL_ (t)	0.04 ± 0	0.72 ± 0.01	1.08 ± 0.01	0.65 ± 0.01	0.03 ± 0.01
ϕ_TL_ (w) [°]	69.3 ± 1.9	81.2 ± 3.2	97.0 ± 4.1	108.9 ± 2.5	115.1 ± 2.3
ϕ_TL_ (t) [°]	69.5 ± 4.4	84.6 ± 3.4	100.7 ± 3.1	111.7 ± 3.6	117.2 ± 2.8
*M*_TL_ (w)	0.005 ± 0.006	−0.007 ± 0.020	−0.083 ± 0.025	−0.106 ± 0.046	0.005 ± 0.014
*M*_TL_ (t)	0.001 ± 0.016	−0.051 ± 0.038	−0.182 ± 0.049	−0.135 ± 0.050	−0.006 ± 0.005
Whippet					
Δl_TL_ (w)	−0.004 ± 0	−0.070 ± 0.014	−0.076 ± 0.017	−0.051 ± 0.017	−0.030 ± 0.016
Δl_TL_ (t)	−0.004 ± 0	−0.073 ± 0.006	−0.080 ± 0.007	−0.032 ± 0.006	0.011 ± 0.003
Fa_TL_ (w)	0.05 ± 0	0.54 ± 0.01	0.58 ± 0.02	0.43 ± 0.02	0.06 ± 0.02
Fa_TL_ (t)	0.04 ± 0	0.67 ± 0.01	0.94 ± 0.01	0.62 ± 0.01	0.04 ± 0
ϕ_TL_ (w) [°]	71 ± 2.7	79.9 ± 3.3	92.9 ± 3.2	103 ± 4	109.2 ± 3.7
ϕ_TL_ (t) [°]	72.2 ± 2.5	83 ± 2.8	96.1 ± 3.8	106.5 ± 4.3	112.2 ± 2.9
*M*_TL_ (w)	0.000 ± 0.012	−0.033 ± 0.032	−0.087 ± 0.035	−0.091 ± 0.037	−0.015 ± 0.006
*M*_TL_ (t)	0.004 ± 0.005	−0.027 ± 0.041	−0.121 ± 0.057	−0.111 ± 0.043	−0.015 ± 0.01

Mean values ±SD, for the axial force (Fa_TL_), the leg length change (Δl_TL_), the angle between leg and trunk vector (ϕ_TL_), and the hip torque (*M*_TL_) at walk (w) and trot (t). Note that Fa_TL_, Δl_TL,_ and *M*_TL_, are dimensionless. *l* is leg length and *l*_0_ leg length at TD; m, mass and g, gravity.

**TABLE 4:** Viscoelastic Parameters for the axial leg.

Breed/Gait	$\hat{k}$ _PL_	$\hat{c}$ _PL_	$\hat{k}$ _TL_	$\hat{c}$ _TL_
Beagle^(1)^/walk	7.4 ± 2.6	−0.6 ± 0.4	9.1 ± 2.7	−0.16 ± 0.4
Beagle^(1)^/trot	7.9 ± 2.1	−1.0 ± 0.5	9.2 ± 1.1	−1.2 ± 0.4
F. Bulldog^(2)^/walk	6.7 ± 3.7	0.2 ± 0.4	6.2 ± 1.6	−0.7 ± 0.6
F. Bulldog^(2)^/trot	7.6 ± 1.1	−0.6 ± 0.3	8.0 ± 1.1	−1.33 ± 0.7
Malinois^(3)^/walk	5.4 ± 1.9	−0.7 ± 0.6	9.3 ± 2.3	−0.6 ± 0.6
Malinois^(3)^/trot	7.2 ± 0.7	−1.0 ± 0.4	9.7 ± 1.9	−1.3 ± 0.4
Whippet^(4)^/walk	7.0 ± 1.6	−0.5 ± 0.7	8.3 ± 1.3	−0.14 ± 0.5
Whippet^(4)^/trot	7.1 ± 0.8	−0.7 ± 0.4	10.3 ± 1.4	−0.9 ± 0.3
Gait	*p* < 0.001 (***)	*p* < 0.001 (***)	*p* < 0.001 (***)	*p* < 0.001 (***)
Breed	*p* = 0.005 (**)	*p* < 0.001 (***)	*p* < 0.001 (***)	*p* < 0.001 (***)
Gait*Breed	*p* = 0.02 (*)	*p* < 0.001 (***)	*p* < 0.001 (***)	*p* = 0.002 (**)
Post-Hoc-Tests				
2\1	n.s.	*p* < 0.001 (***)	*p* < 0.001 (***)	*p* < 0.001 (***)
3\1	*p* = 0.001 (**)	n.s.	n.s.	*p* = 0.001 (**)
4\1	n.s.	n.s.	n.s.	*p* = 0.002 (**)
3\2	n.s.	*p* < 0.001 (***)	*p* < 0.001 (***)	n.s.
4\2	n.s.	*p* < 0.001 (***)	*p* < 0.001 (***)	*p* < 0.001 (***)
4\3	*p* = 0.005 (**)	*p* = 0.048 (*)	n.s.	*p* < 0.001 (***)

$\hat{k}$ : dimensionless stiffness. $\hat{c}$ : dimensionless damping. Values displayed are mean ± SD.

Negative $\hat{c}$ values indicate leg lengthening and energy generation.

### 3.5 Thoracic limb and tangential leg function

As for the pelvic limbs, the thoracic limbs of all breeds touched down with a mean angle of attack ϕ_0_ of approximately 70° at walk. Maximal mean retraction angles ϕ_PL_ were approximately 115° for Beagles and Malinois, 109° for Whippets, and ∼107° for French Bulldogs (Table 3). The torque pattern displayed by all breeds was biphasic. However, the first retraction phase was rather short. It follows protractor torque until TO (Toe off). The work exerted on the scapulothoracic joint was negative (see Figure 3D).

At trot, the leg touched the ground at steeper angles than those observed for walk. However, differences are not significant (for gait, breed, or gait * breed *p* > 0.05). Except for the Beagle, the other three breeds retracted their thoracic limb during the stance phase at trot more than at walk (Table 3).

Mean torque profiles look similar among breeds (Figures 3D, 4D). The first positive half sinus (protractor torque) observed at walk almost disappeared at trot. Therefore, all exerted tangential work was negative. Interestingly, Whippets showed a mean negative maximum torque, which was like the maximum torque exerted at walk. For the other three breeds, peak negative torque at trot was twice as large as at walk.

### 3.6 Thoracic limb and VPP

The position of the thoracic limbs’ VPP (VPP_TL_) is gait and breed-related, but the linear combination gait * breed was not significant. The VPP_TL_ was placed significantly (*p* < 0.001) higher at walk (for Whippets, Malinois, and Beagles approximately 0.7, while for French Bulldogs 1.14 of effective leg length at TD) compared to trot (all breeds showed different distances). Post-hoc test revealed that only the VPP_TL_ obtained for the French Bulldogs was significantly different from the other breeds. The horizontal distance between VPP_TL_ and scapulothoracic joint did not significantly vary between breeds nor did the linear combination gait effects * breed effects (see Table 5). Scatter plots revealed a negative linear relationship between leg length and the vertical position of the VPP_TL_ at trot, while the horizontal position of the VPP_TL_ did no display breed or gait dependencies (Figure 5).

**TABLE 5:** VPP distances to the proximal joints.

Breed/Gait	dVPP_PL_ [% *l*_0_]	dhVPP_PL_ [% *l*_0_]	dVPP_TL_ [% *l*_0_]	dhVPP_TL_ [% *l*_0_]
Beagle^(1)^/walk	0.44 ± 0.14	−0.19 ± 0.09	0.70 ± 0.08	0.15 ± 0.06
Beagle^(1)^/trot	0.24 ± 0.08	−0.06 ± 0.03	0.48 ± 0.18	0.23 ± 0.03
F. Bulldog^(2)^/walk	0.99 ± 0.17	−0.48 ± 0.12	1.14 ± 0.16	0.11 ± 0.12
F. Bulldog^(2)^/trot	0.25 ± 0.12	−0.18 ± 0.07	0.89 ± 0.3	0.23 ± 0.09
Malinois^(3)^/walk	0.92 ± 0.36	−0.19 ± 0.02	0.7 ± 0.19	0.14 ± 0.07
Malinois^(3)^/trot	0.2 ± 0.1	−0.04 ± 0.06	0.19 ± 0.12	0.16 ± 0.04
Whippet^(4)^/walk	0.81 ± 0.2	−0.09 ± 0.10	0.70 ± 0.16	0.26 ± 0.13
Whippet^(4)^/trot	−0.03 ± 0.2	0.01 ± 0.04	0.30 ± 0.05	0.15 ± 0.08
Gait	*p* < 0.001 (***)	*p* < 0.001 (**)	*p* < 0.001 (***)	n.s.
Breed	n.s.	*p* < 0.01 (**)	*p* < 0.001 (***)	n.s.
Gait*Breed	n.s.	n.s.	n.s.	n.s.
Post-Hoc-Tests				
2\1		*p* = 0.03 (*)	*p* < 0.001 (***)	
3\1		n.s.	n.s.	
4\1		n.s.	n.s.	
3\2		*p* < 0.01 (**)	*p* < 0.001 (***)	
4\2		*p* < 0.01 (**)	*p* < 0.001 (***)	
4\3		n.s.	n.s.	

dVPP_PL_ (vertical distance VPP- hip, joint), dhVPP_PL_ (horizontal distance VPP- hip, joint).

dVPP_TL_ (vertical distance VPP- scapulothoracic joint) and dhVPP_TL_ (horizontal distance VPP- scapulothoracic joint) are dimensionless (% *l*_0_). Negative dhVPP_PL_, values indicate that the VPP_PL_, is located cranially to the hip joint.

Positive dhVPP_TL_, values indicate that the VPP_TL_, is located caudally to the scapulothoracic joint.

## 4 Discussion

In the present work, we analyzed dog global dynamics at walk and trot based on kinematic and single-leg kinetic data recorded from French Bulldogs, Whippets, Malinois, and Beagles. Because the four breeds analyzed differ in body proportions, mass, posture, and breed purpose, our results may inform on general principles of dog quadrupedal locomotion. We focused our work on the level of the effective leg. We analyzed both the axial and the tangential effective leg functions and whether those leg functions are related to each other via a local VPP. Note that neither current literature nor this study can confirm that a VPP represents a goal of the motor control system, especially in quadrupeds. However, numerical experiments that used the VPP as a control target may help to understand the relationships between the effective leg and position of VPP.

This study confirms the 45-year-old result of Jayes and Alexander (1978), who found the same two points in about the same position, which today we call VPP. Our work shows additional gait and leg length-related differences in the position of both pelvic and thoracic VPPs. This contradicts (Maus et al. (2010), who presented just one VPP for both pairs of legs. During walk, the VPP of the thoracic limbs (VPP_TL_) is located above and caudally of the scapulothoracic joint, while the VPP of pelvic limbs, VPP_PL_, is located above and cranially of the hip (Figure 2). As hypothesized, during trot, the distance from the VPPs to the proximal joints and the variability between and within breeds tended to lessen compared to walk. But, while the VPP_TL_ remained above the scapulothoracic joint in all dogs, the VPP_PL_ descended closely below the hip for three Whippets and one Malinois. The horizontal distance of the VPP to the pelvis (dhVPP_PL_) was also decreased, while dhVPP_TL_ was mostly increased.

In simulations, the horizontal positions of both VPPs related to their proximal joints largely influenced both axial and tangential leg functions and leg work (Andrada et al., 2014; Blickhan et al., 2015). When the VPP was located above the proximal pivot as observed in human walking, the effective leg displayed a rather symmetric kinematic behavior (similar inner leg angles at TD and TO), together with symmetric vertical GRF and protraction and retraction torques patterns. For such a configuration, a simple spring-like axial leg behavior together with VPP control is able to generate steady-state locomotion (Maus et al., 2010). When the VPP was located cranially to the proximal pivot, as known from birds or Japanese macaques and dog pelvic limbs, the torque generated by directing the GRF to it generated only or mostly positive work in the most proximal joint. Accordingly, birds and Japanese macaques use spring-damped effective leg functions to axially absorb energy and stabilize locomotion (Andrada et al., 2014; Andrada et al., 2015b; Blickhan et al., 2018). Interestingly, dogs with the only exception of French Bulldogs during walk displayed a different strategy. They extended their pelvic limbs during stance, indicating that energy was added to the system. Finally, if the VPP is placed caudally to the proximal pivot, as depicted in the dogs’ thoracic limb, the torque generated will absorb energy. In this case, the leg must add energy axially to the system for the sake of periodicity (Blickhan et al., 2015; Drama and Badri-Spröwitz, 2020). The dogs under study, except Whippets during walk, added energy axially to the system. Interestingly, independently of whether the axial leg was absorbing or generating energy, the axial force varied nearly linear with $∆\dot{\hat{l}}$ . This made it reasonable to model the axial leg function as a spring-damper system using positive or negative damping values (see Figures 3I and 4I and additional discussions on negative damping in Section 4.3).

Our findings contrast to some degree with other simple models based on spring-like or spring-dampened legs (McMahon, 1985; Nanua, 1992; Nanua and Waldron, 1995; Herr and McMahon, 2001; Poulakakis et al., 2003; Poulakakis et al., 2006) and a well-accepted hypothesis like the strut limbs proposed by Carrier and colleagues (Carrier et al., 2005; Carrier et al., 2008).

Based on the principle that neuronal control is rather conservative, we hypothesized that locomotion control principles at the global level should be roughly similar among different dog breeds at the same gait. Our results could not falsify the above-defined hypothesis and seem to support this idea. In general, gait changes or leg length influenced the position of the local VPPs and the patterns of axial and tangential patterns more significantly than the breed.

### 4.1 Pelvic limb control

Mean leg stiffness between breeds oscillated around $\hat{k}$ = 7 for both walk and trot; the only exception was found for Malinois at walk, $\hat{k}$ ≈ 5.5. Mean negative damping increased from walk to trot, mirroring the larger leg lengthening observed at trot for all breeds.

Our results indicate that the distance between the pelvis and VPP_PL_ is gait and leg length related (Figure 5). During walk and trot, the VPP is located cranially to the hip (Figure 2). Its distance to the hip was reduced in trotting dogs, in accordance with a reduction of limb work. French Bulldogs exhibit significantly more cranially located VPP_PL_ during both walk and trot, which seems to be related to their shorter legs (Figure 5C). This position is consistent with only extensor torques in the hip and leg shortening during stance in contrast to the sinus pattern (extension-flexion) for the hip torque and leg lengthening observed for the other breeds with longer legs in this study. Note that the latter patterns are also considered to be a general feature in dogs (Ragetly et al., 2010; Headrick et al., 2014) and in other quadrupeds during level locomotion (Witte et al., 2002; Andrada et al., 2013). French Bulldogs display unusual pelvic limb three-dimensional kinematics as femoral abduction (>40°) and external rotation (>30°) during walk and trot (Fischer et al., 2018). This complex segmental kinematics may additionally hamper leg lengthening, and therefore propulsion is only produced via hip torque. In addition, French Bulldogs have a more cranially located CoM due to their relatively big head and lack of tail. Accordingly, they exhibit lower pelvic vertical GRF/BW when compared to the other breeds analyzed in this study (see Figure 2; Supplementary Figure S5). This fact may permit them to exhibit a more cranially located VPP_PL_ without significantly increasing hip torque, as shown in this work. A more cranially and higher located VPP_PL_ may emerge as means of a control system bound for faster and more powerful movements also in non-sagittal directions, e.g., during a fight. In contrast, a more aligned and closer VPP_PL_ to the hip may display an optimum for striding locomotion. Accordingly, the Malinois and the Whippets display more aligned VPP_PL_ positions related to the hip. Finally, the cranial position of the VPP_PL_ reflects the accelerating fore-aft GRF widely observed in quadruped locomotion (see also Figure 2; Supplementary Figures S3–S6). These accelerating forces are rotating the GRF to the more cranially located VPP_PL_ (see Figure 2).

### 4.2 Thoracic limb control

The thoracic limbs were found to be stiffer than the pelvic limbs during both walk and trot. A stiffer leg function in the thoracic limb may compensate for approximately 50% higher vertical forces compared to the pelvic limbs to maintain similar heights in the hip and scapulothoracic joint. From walk to trot, all breeds increased $\hat{k}$ , but changes were smaller in Beagles and Malinois. These two breeds exhibited a relatively stiffer thoracic limb already at walk. Further walk analyses at lower speeds are necessary to confirm our findings as a common feature in Beagles and Malinois or to unravel a speed-related behavior.

Beagles and Whippets displayed at walk damping coefficients values oscillating around $\hat{c}$ = 0 (spring-like behavior). The confidence intervals in Figure 4H display that during walk, the axial leg function can dissipate or add energy to the system. This finding may reflect an active mechanism to cope with treadmill speed. At trot, the strategy is just one for all breeds (negative damping), mirroring a more automated gait.

The vertical distance to the VPP_TL_ from the scapulothoracic joint (dVPP_TL_) was influenced by gait and leg length. This distance is diminished almost linearly with *l*_0_ during trot, which is likely to reduce joint torques and joint work as GRF increases. Regression lines predict dVPP_TL_ = 0 for *l*_0_ ≈ 0.6 m (Figure 5B), indicating that VPP_TL_ would slide below the scapulothoracic joint for *l*_0_ > 0.6 m. A second, perhaps more likely option, would be that VPP_TL_ approaches asymptotically the scapulothoracic joint for *l*_0_ > 0.6 m.

Independent of gait, the VPP_TL_ is placed caudally to the scapulothoracic joint. This finding indicates that energy absorption tangentially in the effective leg is a mandatory feature in quadrupedal locomotion. The more caudal position of the VPP_TL_ reflects the rather braking anterior-posterior GRF observed during most of the stance phase in dogs’ thoracic limbs (Figure 2; Supplementary Figures S3–S6) (Budsberg et al., 1987; Riggs et al., 1993; Lee et al., 1999; Bertram et al., 2000; Fischer and Lilje, 2011; Andrada et al., 2013). While at TD and in the early support, the leg placement adds to the braking fore-aft forces, during most of the stance, the protractor torque in the proximal pivot (necessary to rotate axial forces to the VPP_TL_) also generates negative fore-aft forces. As our results show, the thoracic limbs work against the retraction of the limb during the stance phase. This fact, which is counter intuitive, explains why the M. latissimus dorsi, the so-called main retractor of the humerus, actually remains silent during striding locomotion in dogs (Carrier et al., 2006; Carrier et al., 2008). Protracting torques in the scapula and/or in the shoulder joints computed via inverse dynamics were previously reported for dogs (Andrada et al., 2017), horses (Clayton et al., 1998; Colborne et al., 1998), small mammals (Witte et al., 2002), and rats (Andrada et al., 2013). The VPP template presented here is a helpful tool to understand such relationships between joint torques and GRFs.

The question is why dogs (and perhaps quadrupeds in general) absorb energy tangentially and add energy axially in their thoracic limbs. The tangential energy absorption may prevent an uncontrolled thoracic leg retraction induced by a more cranially located CoM. The large negative work produced tangentially is then partially compensated by leg lengthening (axial positive work) during stance. This compensation is more marked during trot, in which Whippets, Malinois, and Beagles axially compensate roughly 50%, and French Bulldogs more than 85% of the negative work absorbed tangentially.

### 4.3 Negative damping in the axial leg function

Negative damping means that the force produced by the damper will act in the same direction as $∆\dot{\hat{l}}$ , adding energy to the system. In our case, a negative damping coefficient implies that energy is added axially to the system. Remember that the effective leg (see Figures 1, 3E, F, 4E, F) can add or dissipate energy in two dimensions: axially (the line between the back paw and hip joint or front paw and scapulothoracic joint) and tangentially (torque in the proximal joint). Hereto, the sum of both works specifies whether the leg is adding or dissipating energy.

The negative damping is only an objective measure of what the work loops display in Figures 3A, B, 4A, B for the axial function. There, the work loops rotate clockwise, which indicates that energy was added axially to the system (effective leg was in addition regularly larger at TO than at TD), and the integral below the curve in those figures indicated in most of the cases positive work (see legends on Figures 3A, B, 4A, B). In the pelvic limbs, the sum of axial and tangential works was positive, while in the thoracic limbs was, in total, negative (even when axially the leg added energy). This happened because of the significant energy absorption that occurred in the scapulothoracic joint (Torque attempted to protract the leg but the leg still retracted, see Figure 4D). Thus, in quadrupeds, pelvic and thoracic limbs behave differently. Those behaviors, as shown in this paper, are related to the positions of the VPPs, which in turn depend on gait and leg length. Basically, positive damping can be induced with passive elements, while for generating negative damping one needs a motor.

### 4.4 Differences to the single bipedal VPP

Quadrupeds do not contend with the same issues of trunk stability as bipeds. Since quadrupeds maintain ground contact at least by two legs during walk and trot, they can counter-balance pitching moments easily. Because of the existence of two VPPs, the GRFs are more displaced from the CoM compared to bipeds. This permits dogs to exert larger pitching moments about the CoM, and at the same time to minimize limb work (see Figure 1).

In humans, a VPP promotes whole-body stability, but it does not stabilize the upper body (Müller et al., 2017). These findings led Müller and colleagues to the assumption that the VPP is not a target variable of the control system. Variations in VPP height were observed in studies on humans walking with different trunk inclinations (Müller et al., 2017), in humans walking and running over visible and camouflaged curbs (Vielemeyer et al., 2019; Drama et al., 2020), and in simulation studies (Lee et al., 2017; Schreff et al., 2023) added to that hypothesis. On the other hand, simulations and experimental studies on birds showed that VPP control in combination with a pronograde trunk stabilizes both trunk and overall locomotion (Andrada et al., 2014; Andrada et al., 2015a; Müller et al., 2017). These findings indicate that the overall body plan influences the stabilizing effect of a VPP.

Our results on dog walk and trot support the idea that the VPP location reflects the “tuning of the whole musculoskeletal system for efficient gait” (Müller et al., 2017). It remains intriguing if and how negative damping (leg axial extension), together with proximal joint work generation/absorption, and VPPs’ position influence trunk and overall quadrupedal locomotion stability.

## Data Availability

The raw data supporting the conclusion of this article will be made available by the authors, without undue reservation.
