# Supplementary material for: Positioning of pivot points in quadrupedal locomotion: limbs global dynamics in four different dog breeds
Source: Front Bioeng Biotechnol. 2023 Jul 7;11:1193177. doi: 10.3389/fbioe.2023.1193177 (PMC10360120; doi:10.3389/fbioe.2023.1193177)
Supplement: Supplementary file 1 [file DataSheet1.pdf]

Positioning of pivot points in quadrupedal locomotion: limbs global dynamics in four different dog breeds.

Emanuel Andrada[1,2], Gregor Hildebrandt[1,2], Hartmut Witte[2], Martin S. Fischer[1]

[1] Institute of Zoology and Evolutionary Research, Friedrich-Schiller-University Jena. Germany.

[2] Group of Biomechatronics, Institute of Mechatronic System Integration, Technische Universität Ilmenau, Germany.

Supplementary material

a) Finding VPP position from a proximal joint:

The Virtual Pivot Point (VPP) is a geometrical place where the sagittal projection of the ground reaction force's (GRF) vectors during a stance phase cross /point. In our paper, we used a similar method as proposed in (Andrada et al.. 2014). However, here the VPP is related to the proximal joints of the pelvic and thoracic limbs. VPP position was defined as the distance from the proximal joint at which the horizontal spread between the GRFs is minimal.

We used the two points form of equation of the line to obtain the equation of the action line of the GRF at every analyzed time-point of the stance phase (starting from the foot. see Fig. S1).

Starting from the equation of the form $y = mx + b$ (eq. 1) and using the coordinates of the proximal joint (pelvis or scapular pivot) as origin (proximal joint-fixed coordinate system), the slope of the equation action line of the GRF can be obtained as:

$$m = \frac{-l_y - F_y - l_y}{-l_x - F_x - l_x}$$

Where l_x and l_y are the horizontal and vertical projections of the effective leg length, and F_x and F_y the horizontal and vertical components of the GRF.

The y-intercept is:

$$b = -l_y - ml_x$$

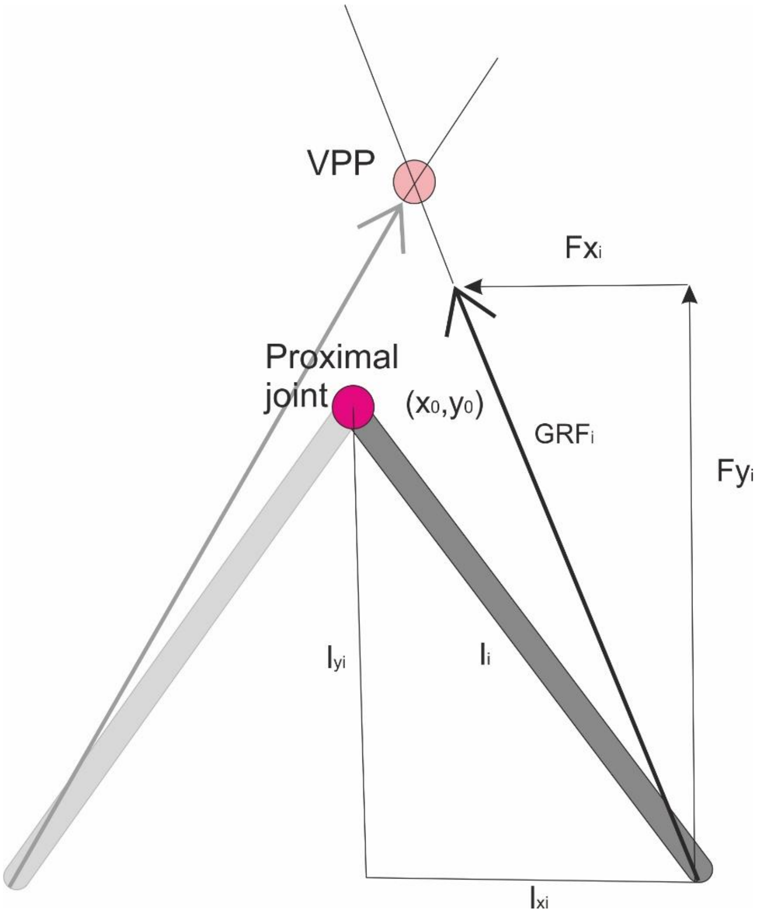

Figure S1: VPP position from a proximal joint.

To obtain x values from eq. 1, we generated a vector containing 50 values for y ranging from -0.1 BW to 2.0 BW. This procedure was repeated from 10% to 90% of the stance in steps of 10 %. Afterwards, mean, and standard deviation for all x values corresponding to a high y were computed. We have chosen as VPP high (y_{VPP}) the y-value at which the standard deviation of x was minimal. The horizontal VPP position (x_{VPP}) is then the mean value of x for y_{VPP}.

b) Processing of the GRF

We collected GRFs at 1,000 Hz. Force data was afterwards down-sampled to 100 Hz to cope with kinematical data and posteriorly filtered using a 7th-order Butterworth low-pass filter with a cut-off frequency of 20 Hz applied in a zero-phase digital filter. An example showing vertical and fore-aft GRFs before and after filtering is presented in the figure S2 (see below).

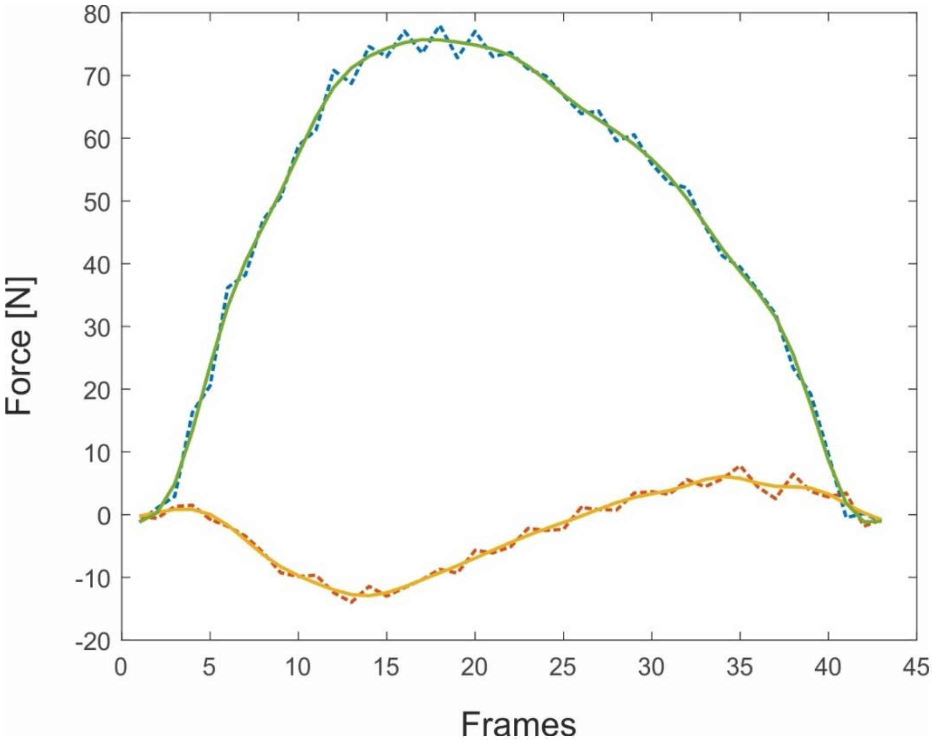

Figure S2: Raw (dashed lines) and filtered (solid lines) vertical and fore-aft components of the GRFs from a thoracic limb of a Beagle.

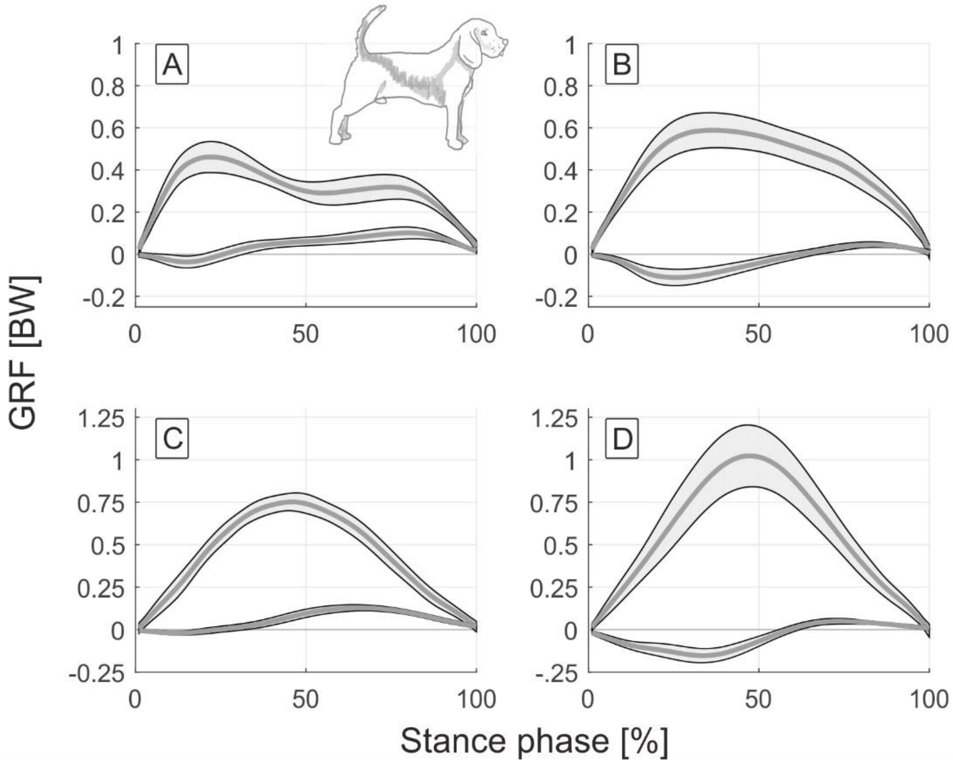

Figure S3: Ground reaction forces in the Beagle during walk (A, B) and trot (C, D). Pelvic limb (A, C), thoracic limb (B, D).

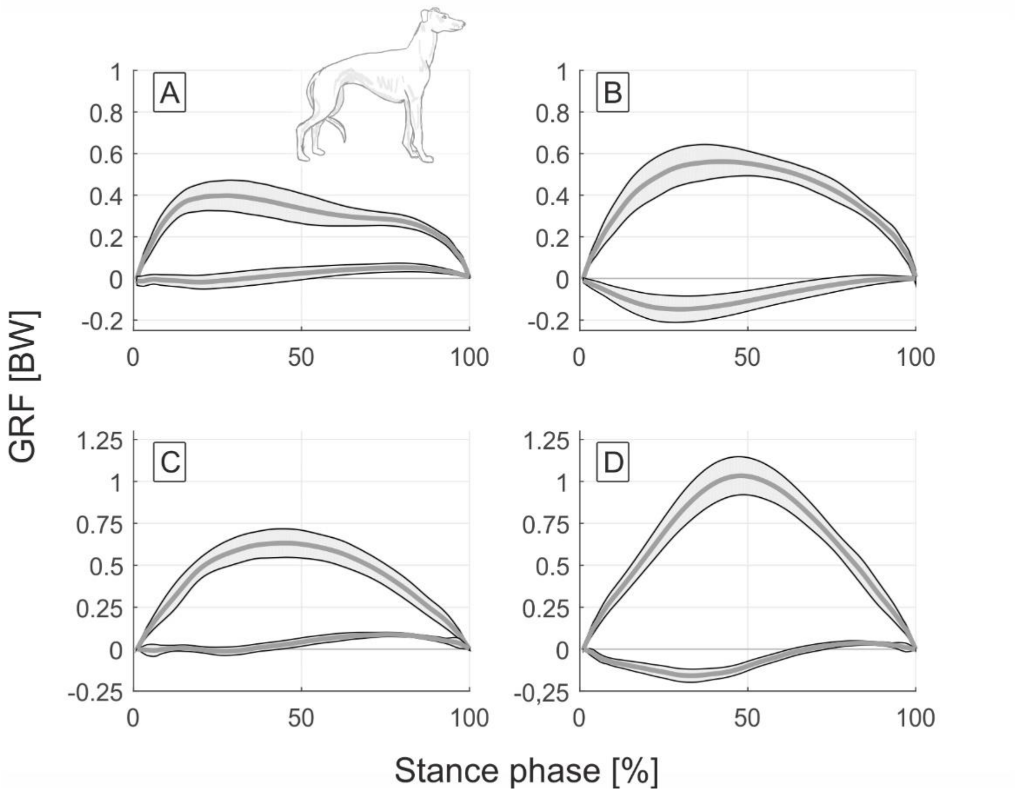

Figure S4: Ground reaction forces in the Whippet during walk (A, B) and trot (C, D). Pelvic limb (A, C), thoracic limb (B, D).

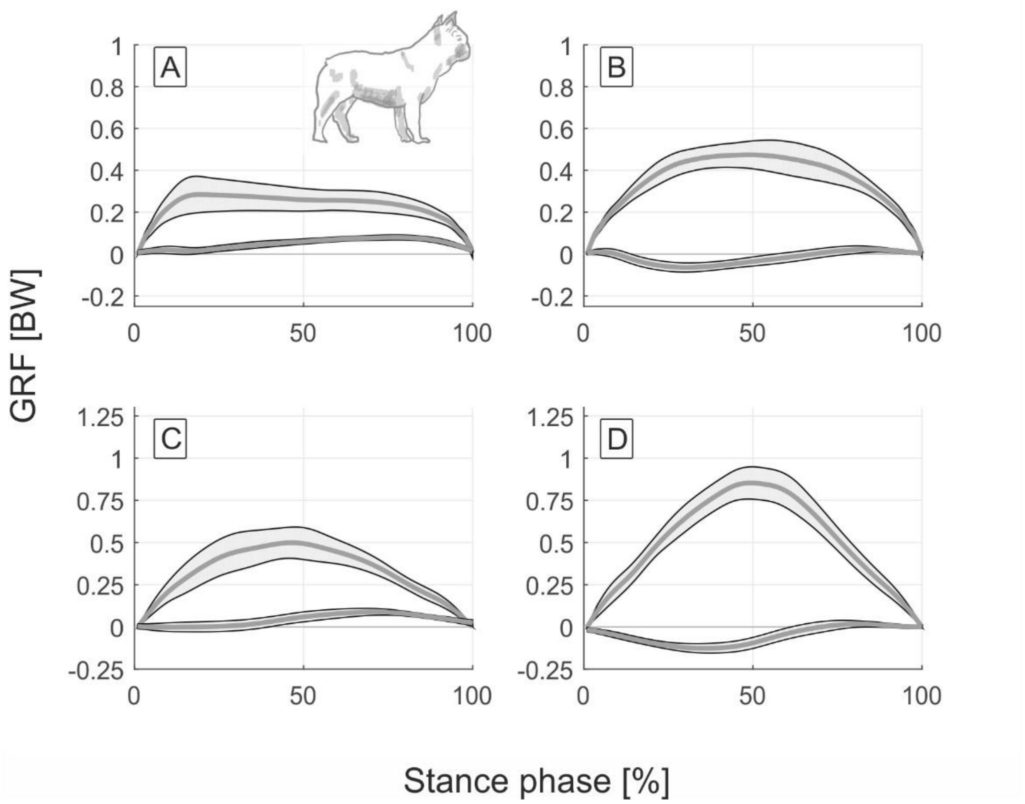

Figure S5: Ground reaction forces in the Bulldog during walk (A, B) and trot (C, D). Pelvic limb (A, C), thoracic limb (B, D).

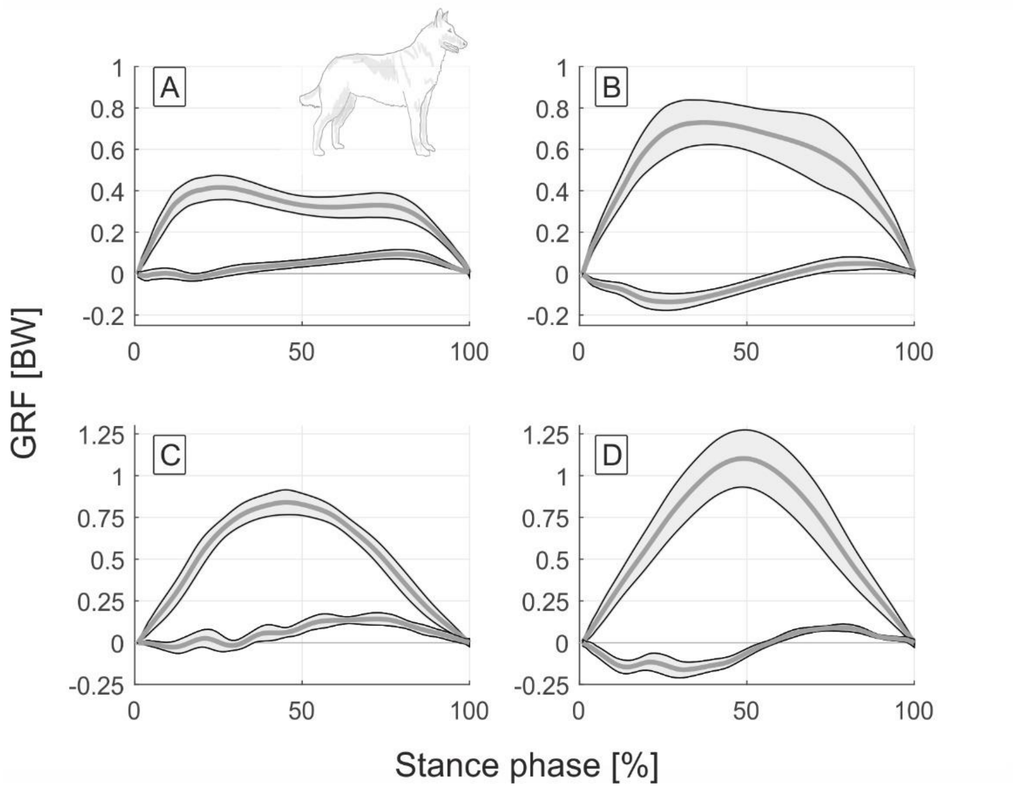

Figure S6: Ground reaction forces in the Malinois during walk (A, B) and trot (C, D). Pelvic limb (A, C), thoracic limb (B, D).

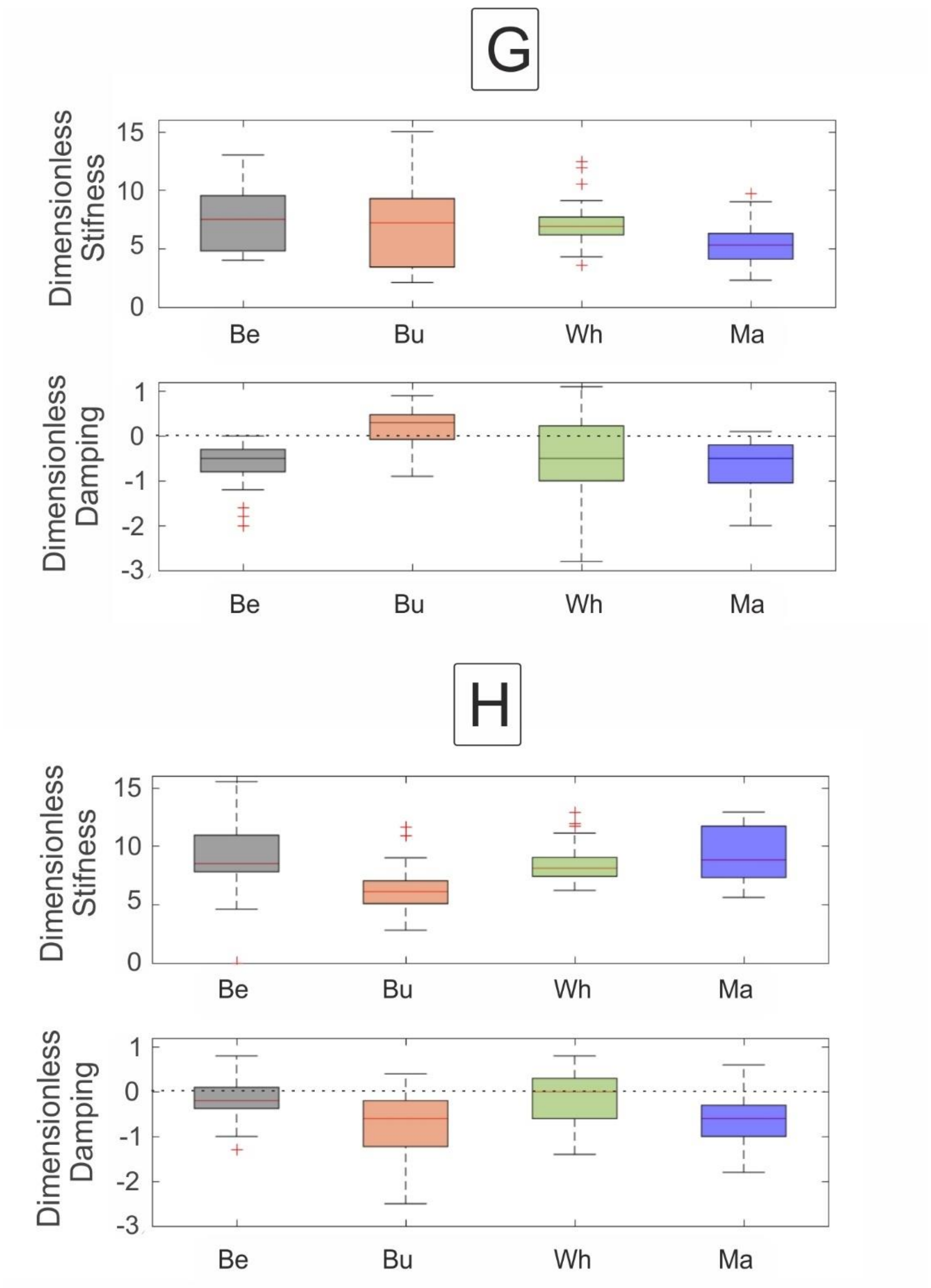

Figure S7: Extract of figures 3G, 3H. Dimensionless stiffness and damping of the effective leg at walk in Beagle (black), Whippet (green), French Bulldog (orange) and Malinois (blue). G: pelvic limb, H: thoracic limb. Values are dimensionless.

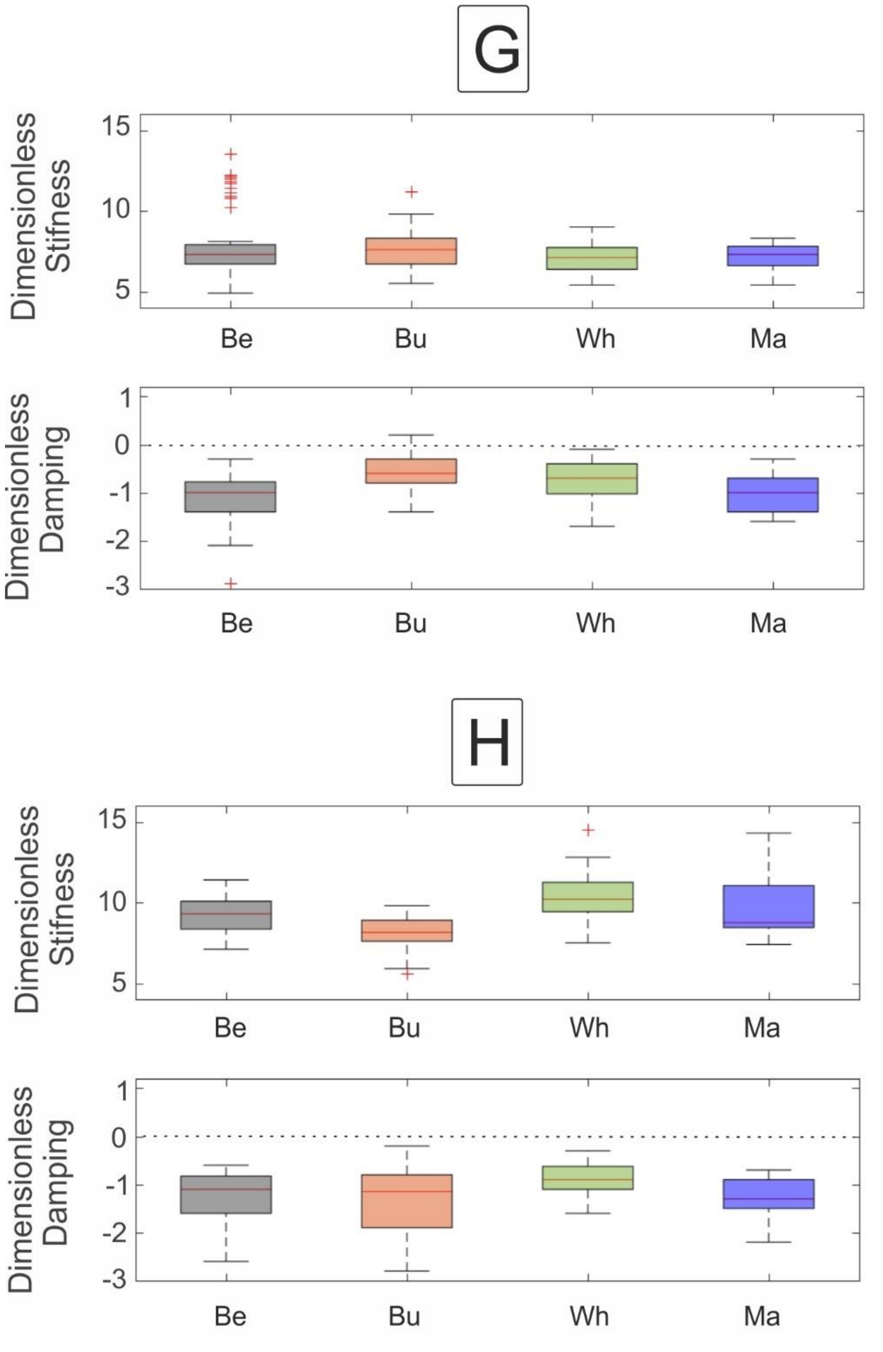

Figure S8: Extract of figures 4G, 4H. Dimensionless stiffness and damping of the effective leg at trot in Beagle (black), Whippet (green), French Bulldog (orange) and Malinois (blue). G: pelvic limb, H: thoracic limb. Values are dimensionless.

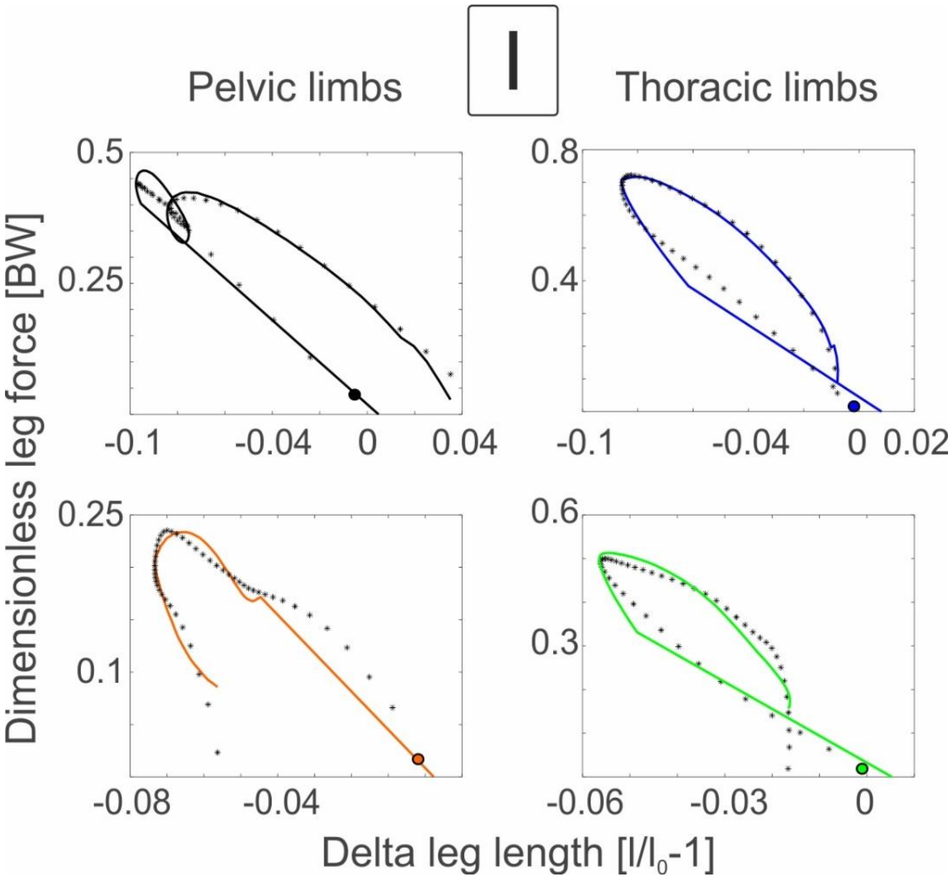

Figure S9: Extract of figure 3I. Examples of axial leg function from experiments at walk (black asterisks) and results of the nonlinear fit using a parallel spring-damper model (colored line). Beagle (black), Whippet (green), French Bulldog (orange) and Malinois (blue). Values are dimensionless.

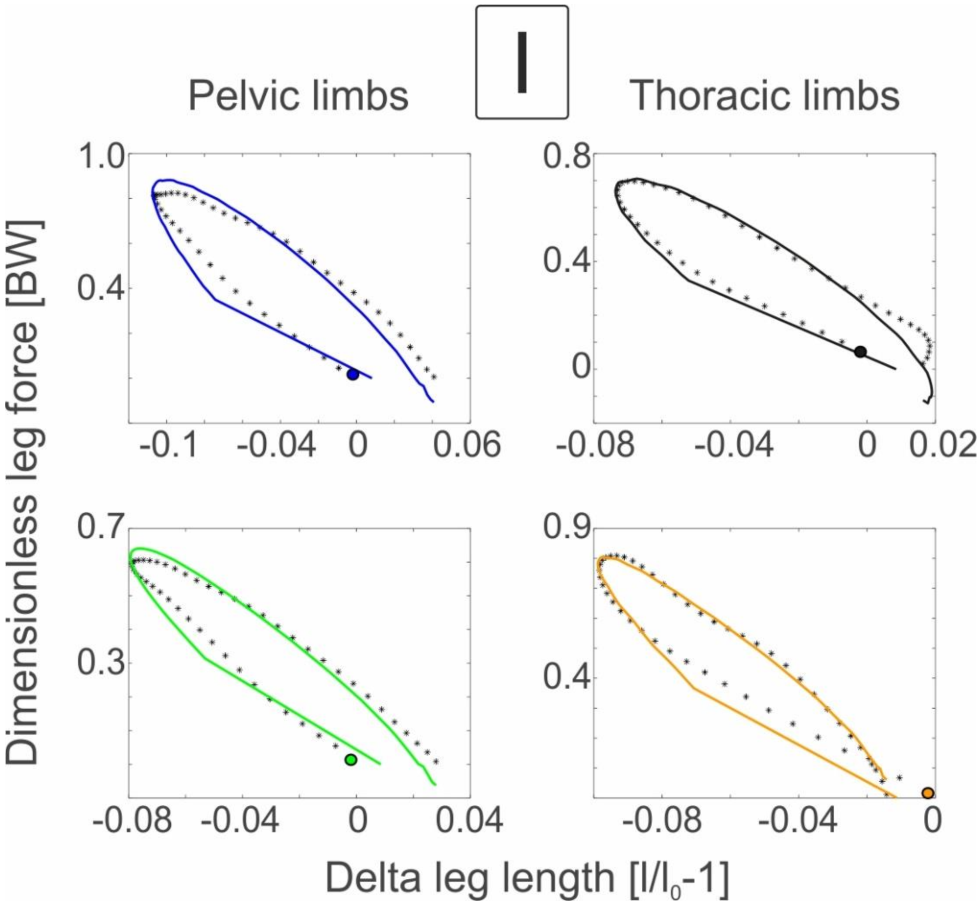

Figure S10: Extract of figure 4I. Examples of axial leg function from experiments at trot (black asterisks) and results of the nonlinear fit using a parallel spring-damper model (colored line). Beagle (black), Whippet (green), French Bulldog (orange) and Malinois (blue). Values are dimensionless.

c) Additional statistical results:

Method: Repeated measures ANOVA with Gait (walk vs. trot) as within subjects and Breed as between subjects.

b1. Maximal leg axial force $F_{a\text{-}max}$ in BW (body weight)

Pelvic Limb

	**Mean $F_{a\text{-}max}$**	**SD (±)**
Beagles_w(1)	0.47	0.08
Beagles_t(1)	0.76	0.04
Bulldogs_w(2)	0.32	0.08
Bulldogs_t(2)	0.53	0.08
Malinois_w(3)	0.42	0.05
Malinois_t(3)	0.85	0.07
Whippet_w(4)	0.41	0.07
Whippet_t(4)	0.64	0.1
Gait	$p < 0.001$(***)	
Breed	n.s.	
Gait*Breed	n.s.	

w: walk. t: trot. n.s.=non-significant. SD: standard deviation

Thoracic Limb

	**Mean $F_{a\text{-}max}$**	**SD (±)**
Beagles_w(1)	0.61	0.09
Beagles_t(1)	1.08	0.16
Bulldogs_w(2)	0.53	0.08
Bulldogs_t(2)	0.92	0.11
Malinois_w(3)	0.76	0.11
Malinois_t(3)	1.11	0.19
Whippet_w(4)	0.62	0.07
Whippet_t(4)	0.97	0.13
Gait	$p < 0.001$(***)	
Breed	n.s.	
Gait*Breed	n.s.	

w: walk. t: trot. n.s.=non-significant. SD: standard deviation

b2. Leg length at toe-off (TO) [$(l-l_0)/l_0$]. l is leg length and l_0 leg length at touch down

Pelvic Limb

	**Mean functional leg length at TO (toe-off)**	**SD (±)**
Beagles_w(1)	0.04	0.03
Beagles_t(1)	0.06	0.01
Bulldogs_w(2)	- 0.03	0.04
Bulldogs_t(2)	0.01	0.01
Malinois_w(3)	0.04	0.03
Malinois_t(3)	0.08	0.02
Whippet_w(4)	0.01	0.03
Whippet_t(4)	0.05	0.02
Gait	p<0.001(***)	
Breed	n.s.	
Gait*Breed	n.s.	

w: walk. t: trot. n.s.=non-significant. SD: standard deviation.

Thoracic Limb

	**Mean functional leg length at TO (toe-off)**	**SD (±)**
Beagles_w(1)	0.00	0.05
Beagles_t(1)	0.03	0.02
Bulldogs_w(2)	- 0.01	0.04
Bulldogs_t(2)	0.03	0.02
Malinois_w(3)	- 0.01	0.02
Malinois_t(3)	0.04	0.01
Whippet_w(4)	- 0.03	0.02
Whippet_t(4)	0.01	0.00
Gait	p<0.001(***)	
Breed	n.s.	
Gait*Breed	n.s.	

w: walk. t: trot. n.s.=non-significant. SD: standard deviation

b.3 Angle of attack [°]

Pelvic Limb

	**Leg angle at TD (touch down) [°]**	**SD(±)**
Beagles_w(1)	68.3	1.3
Beagles_t(1)	73.3	1.6
Bulldogs_w(2)	70.2	3.8
Bulldogs_t(2)	72.3	3.2
Malinois_w(3)	70.2	3.8
Malinois_t(3)	73.3	3.2
Whippet_w(4)	72.1	3.1
Whippet_t(4)	74.9	2.0
Gait	p<0.001(***)	
Breed	n.s.	
Gait*Breed	n.s.	

w: walk. t: trot. n.s.=non-significant. SD: standard deviation

Thoracic Limb

	**Leg angle at TD (touch down) [°]**	**SD(±)**
Beagles_w(1)	69.4	3.0
Beagles_t(1)	73.3	4.9
Bulldogs_w(2)	67.5	5.0
Bulldogs_t(2)	67.7	4.9
Malinois_w(3)	69.0	1.9
Malinois_t(3)	68.9	4.4
Whippet_w(4)	70.7	2.8
Whippet_t(4)	71.7	2.5
Gait	n.s.	
Breed	n.s.	
Gait*Breed	n.s.	

w: walk. t: trot. n.s.=non-significant. SD: standard deviation

b.4 Maximal dimensionless torque ($\hat{T} = T/mgl_0$). l_0 = leg length at touch down, m = mass, and g = gravity.

Pelvic Limb

	Maximal torque	SD(±)
Beagles_w(1)	0.09	0.04
Beagles_t(1)	0.03	0.04
Bulldogs_w(2)	0.04	0.11
Bulldogs_t(2)	0.07	0.01
Malinois_w(3)	0.10	0.03
Malinois_t(3)	0.01	0.09
Whippet_w(4)	0.06	0.08
Whippet_t(4)	0.02	0.08
Gait	p<0.05(*)	
Breed	n.s.	
Gait*Breed	n.s.	

w: walk. t: trot. n.s.=non-significant. SD: standard deviation

Thoracic Limb

	Maximal torque	SD(±)
Beagles_w(1)	0.07	0.08
Beagles_t(1)	0.22	0.06
Bulldogs_w(2)	0.04	0.10
Bulldogs_t(2)	0.17	0.09
Malinois_w(3)	0.11	0.05
Malinois_t(3)	0.19	0.05
Whippet_w(4)	0.10	0.04
Whippet_t(4)	0.13	0.06
Gait	p<0.001(***)	
Breed	n.s.	
Gait*Breed	n.s.	

w: walk. t: trot. n.s.=non-significant. SD: standard deviation

literature:

Andrada. E., Rode. C., Sutedja. Y., Nyakatura. J. A. and Blickhan. R. (2014). Trunk orientation causes asymmetries in leg function in small bird terrestrial locomotion. *Proceedings of the Royal Society B: Biological Sciences* **281**.
